# Seascape genomics of common dolphins (*Delphinus delphis*) reveals adaptive diversity linked to regional and local oceanography

**DOI:** 10.1186/s12862-022-02038-1

**Published:** 2022-07-12

**Authors:** Andrea Barceló, Jonathan Sandoval-Castillo, Chris J. Brauer, Kerstin Bilgmann, Guido J. Parra, Luciano B. Beheregaray, Luciana M. Möller

**Affiliations:** 1 Molecular Ecology Laboratory, College of Science and Engineering, Flinders University, Bedford Park, SA Australia; 2 Cetacean Ecology, Behaviour, and Evolution Laboratory, College of Science and Engineering, Flinders University, Bedford Park, SA 5042 Australia; 3 School of Natural Sciences, Macquarie University, Sydney, NSW Australia

**Keywords:** Delphinids, Adaptive resilience, ddRAD-seq, Landscape genomics, Conservation genomics

## Abstract

**Background:**

High levels of standing genomic variation in wide-ranging marine species may enhance prospects for their long-term persistence. Patterns of connectivity and adaptation in such species are often thought to be influenced by spatial factors, environmental heterogeneity, and oceanographic and geomorphological features. Population-level studies that analytically integrate genome-wide data with environmental information (i.e., seascape genomics) have the potential to inform the spatial distribution of adaptive diversity in wide-ranging marine species, such as many marine mammals. We assessed genotype-environment associations (GEAs) in 214 common dolphins (*Delphinus delphis*) along > 3000 km of the southern coast of Australia.

**Results:**

We identified 747 candidate adaptive SNPs out of a filtered panel of 17,327 SNPs, and five putatively locally-adapted populations with high levels of standing genomic variation were disclosed along environmentally heterogeneous coasts. Current velocity, sea surface temperature, salinity, and primary productivity were the key environmental variables associated with genomic variation. These environmental variables are in turn related to three main oceanographic phenomena that are likely affecting the dispersal of common dolphins: (1) regional oceanographic circulation, (2) localised and seasonal upwellings, and (3) seasonal on-shelf circulation in protected coastal habitats. Signals of selection at exonic gene regions suggest that adaptive divergence is related to important metabolic traits.

**Conclusion:**

To the best of our knowledge, this represents the first seascape genomics study for common dolphins (genus *Delphinus*). Information from the associations between populations and their environment can assist population management in forecasting the adaptive capacity of common dolphins to climate change and other anthropogenic impacts.

**Supplementary Information:**

The online version contains supplementary material available at 10.1186/s12862-022-02038-1.

## Background

Microevolutionary processes influenced by environmental heterogeneity can create adaptive divergence among populations [1–3]. Marine ecosystems are environmentally heterogeneous, with coastal and pelagic species impacted by contrasting selective pressures that can lead to local adaptation (e.g., [3–5]). Local adaptation occurs when an individual or group of individuals display higher fitness in a distinct spatial and temporal environment due to specific genetic variants [6–8]. Natural selection acts on both new mutations and standing genetic variation, with most adaptations involving multiple loci and genomic regions [9, 10]. In marine environments, understanding the influence of geomorphological and oceanographic features, as well as anthropogenic pressures on genomic variation, enhances our ability to refine knowledge about population structure and rapid evolution [11, 12], including for widespread species with high dispersal potential.

Genomic data has improved our understanding of macro- and microevolutionary processes, providing greater power and accuracy to detect large scale molecular adaptations, as well as population structure, gene flow and adaptive divergence between populations [13–15]. In toothed whales (Odontoceti), most studies of adaptations using genomic markers have focused on a macroevolutionary perspective, while studies investigating ecological specialisation on a microevolutionary level have been documented only for a few species. This includes ecotype adaptations of killer whales (*Orcinus orca* [16]), spinner dolphins (*Stenella longirostris* [17]), finless porpoises (*Neophocaena phocaenoides* [18, 19]), and bottlenose dolphins (*Tursiops aduncus* [20], and *T. truncatus* [21]). Despite these examples, population-level studies of microevolutionary processes remain highly under documented in Odontocetes, specifically in small cetaceans. In particular, little is known about the adaptive resilience of small cetaceans to local or regional environmental changes and to future climatic scenarios. The lack of such studies constrains our capacity to provide information for conservation and management, as well as clarifying important aspects of a species’ biology.

The common dolphin (*Delphinus delphis*) is a widespread small cetacean that inhabits temperate, subtropical and some tropical waters around the world [22–24]. Their broad distribution suggests that several habitats are suitable for this species (e.g., [25, 26]). In Australia, common dolphins range from embayments and gulf waters to coastal, shelf and pelagic waters [26–28]. From a neutral genomic perspective, the species in Australasia displays a hierarchical metapopulation structure and fine-scale population sub-structuring [29]. Although common dolphins exhibit high potential for dispersal, prey distribution has been suggested as a main driver for their movements [27, 30, 31]. In Australasian waters, they mainly hunt and feed upon schooling fish such as jack mackerel (*Trachurus declivis, T. s. murphyi* and *T. novaezelandiae*), blue mackerel (*Scomber australasicus*), sardines (*Sardina sagax*) and anchovies (*Engraulis australis*) [32, 33]. The ranges of common dolphin populations seem to be influenced by the distribution and abundance of their prey, and often coincide with oceanographic circulation, areas of high primary productivity, and regions of high salinity and low sea surface temperature interfaces [27, 31, 34]. This suggests that oceanographic features and oceanic circulation patterns could be shaping dispersal of common dolphins, as described for other Australian marine taxa (e.g., [35–37]). However, associations between environmental variables and genetic populations of common dolphin have only been described at broad geographical scales between different oceans [34], and the impact of regional oceanographic features is yet to be revealed. Common dolphins in southern Australia are subject to various anthropogenic stressors, such as interactions and mortalities in fisheries (e.g., [28, 38, 39]), and climatic change (e.g., [40, 41]), both of which can lead to negative health outcomes and potential declines of populations (e.g., [42, 43]). The widespread distribution of common dolphins in southern Australia, where marine environmental gradients and discontinuities are observed, provides an excellent opportunity to investigate microevolutionary processes and adaptive divergence in a highly-mobile marine species.

The temperate waters of southern Australia harbour productive habitats for common dolphins (e.g., [31, 38, 44]). Australia’s southern zonal coastal boundary stretches for > 3000 km, with high species endemism [45]. The geographic discontinuity along the large extent of the southern coastal and shelf waters is mainly characterised by: (1) geological features such as > 400 canyons, one bight of > 1000 km, two large inverse estuaries, and a shallow strait; and (2) oceanographic features such as complexly variable bathymetry, strong currents, presence of seasonal upwellings, and gradients in current velocity, salinity and temperature (e.g., [46–48]). These features, which vary from west to east, have been reported to influence historical genetic and genomic subdivision of invertebrate and fish species (e.g., *Nerita* spp. and *Siphonaria* spp. [49], *Chlorophyta* spp., *Phaeophyta* spp. and *Rhodophyta* spp. [50], and *Catomerus polymerus* [51]). Oceanographic and geological characteristics also impact different plankton biomasses (e.g., [52]), which small pelagic fish feed upon [46]. In turn, restrictions in the distribution of these primary producers and consumers may indirectly impact the genetic variation and adaptive potential of large marine predators, including common dolphins, which feed upon small pelagic species across the region.

Seascape genetic/genomics assessments combining genotype and environment associations in marine systems, have the potential to clarify the relative influence of environment and space on genomic variation [3, 53, 54]. Previous studies on population genetic structure of common dolphins in southern Australia based on neutral markers (e.g., mtDNA, microsatellites) [28, 31] and putatively neutral SNPs [29] have hypothesised that common dolphin populations are associated with environmental gradients. For other marine species, seascape genomic/genetic analyses have suggested that adaptive population structure may be driven by environmental gradients of bathymetry, temperature, oxygen, and salinity (e.g., [53, 55]), while for common dolphins, associations with temperature and chlorophyll have been proposed at the scale of oceanic basins [34].

In this seascape genomics study, genome-wide and environmental data were used to identify loci under selection, and to assess putatively adaptive population structure and diversity along southern Australia. We hypothesised that the continuous distribution of common dolphins in the highly heterogeneous coast of southern Australia influences the genomic variation of the species across different bioregions, leading to adaptive divergence among populations associated with geological and oceanographic features. Findings here can inform about the number and distribution of common dolphin populations and assist with the conservation and management of the species across the region, where it is subject to fisheries interactions, and other anthropogenic impacts such as pollution and climate change (e.g., [38, 56]).

## Results

### Population genomic dataset

**Fig. 1:**
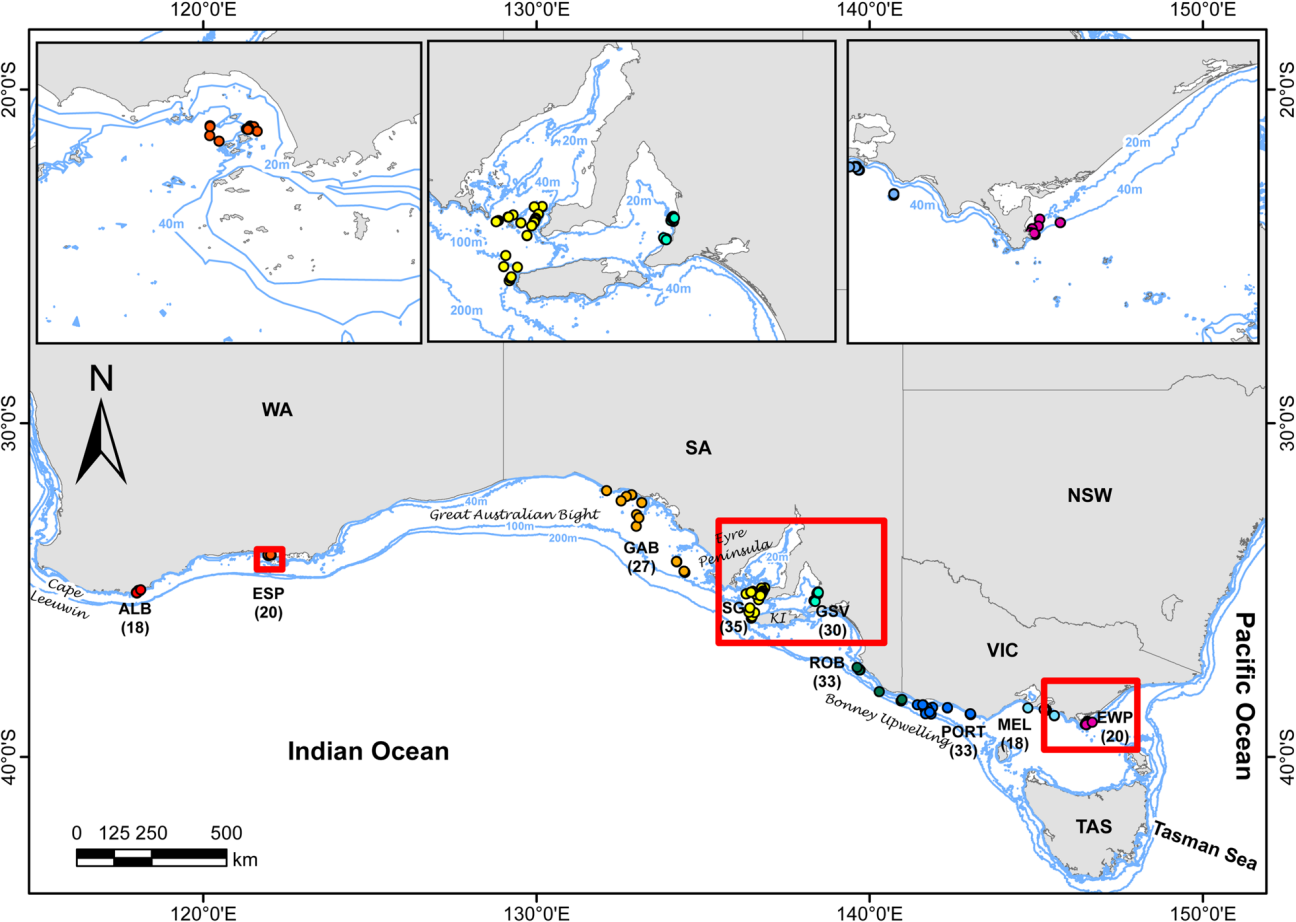
Study area in southern Australia showing the geolocations of 234 common dolphins (*D. delphis*) biopsy sampled for the genomic analyses. *Acronyms: Albany (ALB); Esperance (ESP); Great Australian Bight (GAB); shelf waters, Spencer Gulf (SG); Gulf St Vincent (GSV); Robe (ROB); Portland (PORT); Melbourne (MEL); and East Wilsons Promontory (EWP); Kangaroo Island (KI)

A total of 234 biopsy samples of common dolphins were used and sequenced across four Illumina HiSeq 2500 lanes, producing 400 million filtered sequence reads. After filtering using stringent criteria (detailed in Additional file 1: Table S1 and [29]), we obtained a high-resolution dataset of 17,875 filtered SNPs with 1% average missing data per locus. Low-quality individuals, replicates and close relatives (|R|≥ 0.5) were removed, resulting in a dataset of 214 individuals for analyses. This dataset was then filtered for Minor Allele Count (MAC) < 3, resulting in a final dataset of 17,327 SNPs (Fig. 1).

### Genotype-environment associations

**Fig. 2:**
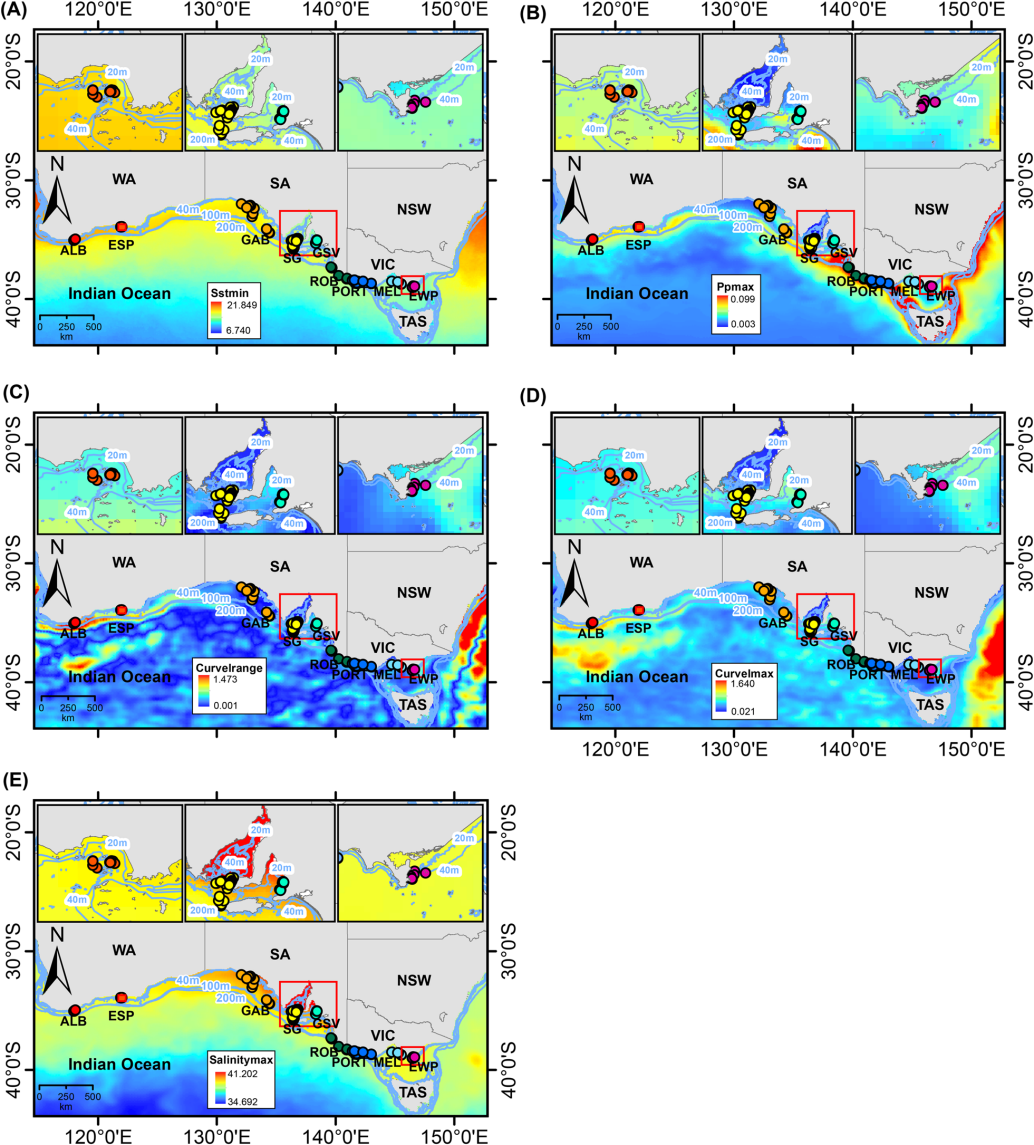
Environmental variables that were retained as significant for the Genotype- Environment and Redundancy Canonical Analyses for southern Australian common dolphins (*D. delphis*). **A** Sea surface temperature minimum, **B** Primary productivity maximum, **C** Current velocity range, **D** Current velocity maximum, and **E** Salinity maximum. *Acronyms as in Fig. 1

A total of six significant MEMs (α < 0.01) were retained and used as spatial variables. Out of 24 environmental variables initially included, five were selected after testing pairwise correlation and multicollinearity (|*r*|> 0.7 and VIF ≥ 3) (Additional file 1: Fig. S1). The retained variables were salinity maximum, sea surface temperature minimum, primary productivity maximum, and current velocity maximum and range, with each environmental variable showing a marked gradient along southern Australia’s coast and shelf waters (Fig. 2a–e).

**Fig. 3:**
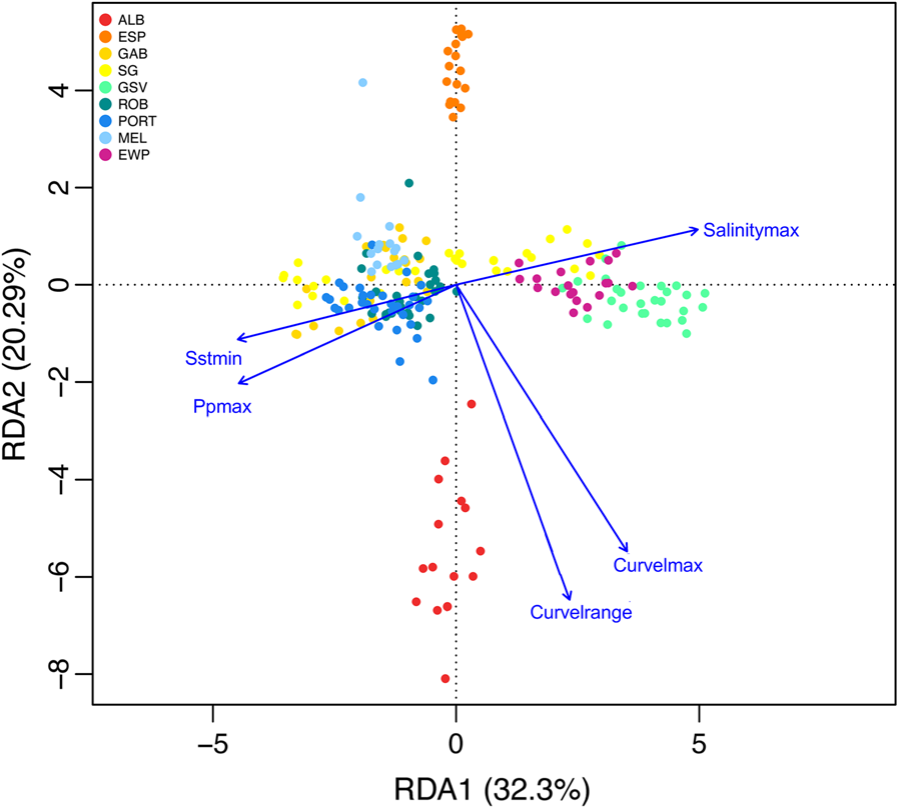
Redundancy Canonical Analysis (RDA) displaying the influence of five environmental variables on individual genomic variation of common dolphins (*D. delphis*) from southern Australia. Legend displays sampling sites from west to east, and colours correspond to where common dolphins were sampled. *Acronyms as in Fig. 1

The overall RDA model was significant (p = 0.001), with the spatial variables explaining 4.9% of the variation of the full model, while the genotype dataset and the environmental variables explained 3.5% of the variation. Each of the five retained environmental variables were significant (p = 0.001) (Additional file 1: Table S3). A total of 747 SNPs were retained as candidate adaptive markers. The first component explained 32% of the constrained variance, while the second component explained 20% of the constrained variance. The RDA biplot demonstrates variation in the genomic response to the five environmental variables among sampling locations (Fig. 3). Changes in primary productivity, sea surface temperature and salinity explained most of the genomic divergence of common dolphins from the Great Australian Bight (GAB), Spencer Gulf (SG), Robe (ROB), Portland (PORT), Melbourne (MEL), East Wilsons Promontory (EWP) and Gulf St Vincent (GSV), with the latter two impacted primarily by higher salinity. In contrast, current velocity variables were strongly associated with the genomic differentiation of common dolphins between the two geographically close sites of Albany (ALB) and Esperance (ESP), Western Australia.

### Adaptive population genomic structure and diversity

The inferred levels of putatively adaptive genome-wide diversity were relatively high for all sites (H_E_ 0.369–0.405; H_O_ 0.361–0.402) compared to the neutral genomic diversity (H_E_ 0.160–0.181; H_O_ 0.154–0.175) previously reported [29] (Table 1).

**Table 1:** Measures of genomic diversity by sampling site based on 747 putatively adaptive SNPs (this study), and 14,799 putatively neutral SNPs (Barceló et al., 2021) from southern Australian common dolphins (*Delphinus delphis*)

Site	N	Neutral		Adaptive	
		H_o_	H_E_	H_o_	H_E_
ALB	15	0.167	0.166	0.399	0.400
ESP	18	0.171	0.172	0.401	0.403
GAB	22	0.170	0.176	0.399	0.416
SG	32	0.172	0.172	0.419	0.419
GSV	28	0.154	0.160	0.380	0.387
ROB	31	0.172	0.175	0.404	0.413
PORT	32	0.169	0.175	0.406	0.417
MEL	16	0.169	0.177	0.395	0.413
EWP	20	0.175	0.181	0.388	0.406
Total average		*0.169*	*0.173*	*0.399*	*0.408*
Total SD		*0.006*	*0.006*	*0.011*	*0.010*

Observed heterozygosity (H_O_), expected heterozygosity (H_E_) and number of samples used after filtering adaptive dataset (N). *Acronyms: Albany (ALB); Esperance (ESP); Great Australian Bight (GAB); shelf waters, Spencer Gulf (SG); Gulf St Vincent (GSV); Robe (ROB); Portland (PORT); Melbourne (MEL); and East, Wilsons Promontory (EWP)

Italics show the overall total and standard deviation across sampling sites

**Fig. 4:**
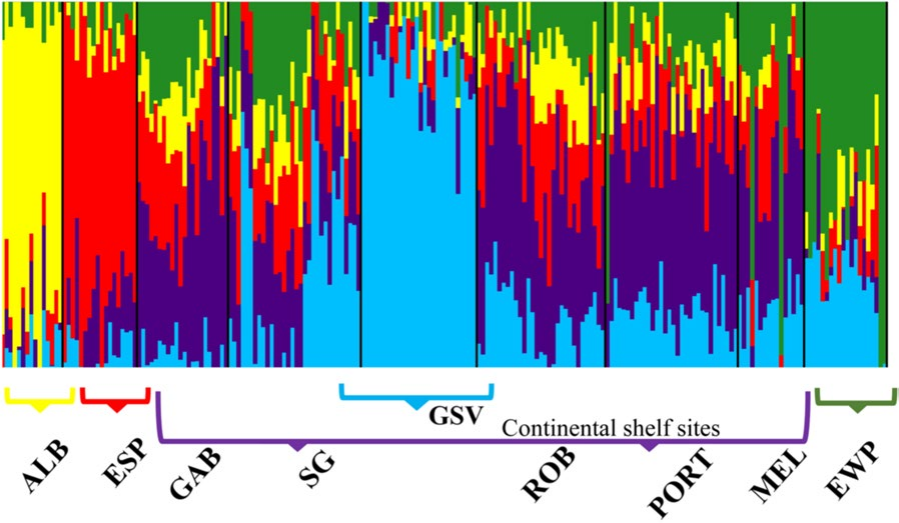
Population genomic structure analysis using Admixture based on 747 putatively adaptive SNPs for southern Australian common dolphins (*D. delphis*), labelled by sampling site. The results depict levels of admixture for each individual sample, grouping them into five adaptive genomic clusters (K = 5). Each sample is represented by one vertical line and is colour-coded based on the membership probability to one of the identified locally adapted populations. *Acronyms as in Fig. 1

Multiple analyses (described below) using the adaptive dataset indicated the presence of four to five local putative populations, which were supported by the AIC test (Additional file 1: Fig. S2): (1) Albany (ALB), (2) Esperance (ESP), (3) Continental shelf sites (GAB, SG, ROB, PORT and MEL), (4) Gulf St Vincent (GSV), and (5) East Wilsons Promontory (EWP). Specifically, Admixture analysis revealed up to five putatively adaptive populations, with a separation between ALB and ESP, GSV, and EWP, compared to considerable admixture among the other sites (Fig. 4; Additional file 1: Fig. S3a–f). PCA results mostly supported four populations, showing in the first two axes only a subtle separation between EWP, GSV sites and [ALB with ESP], with admixed individuals from ALB, ESP and the continental shelf sites clustering in the middle of the two axes, while the third axis show support for a subtle separation between sites of ALB and ESP, with the latest showing a closer association with continental shelf sites (Additional file 1: Fig. S4a–c).

Fixation indices indicated low to moderate (F_ST_ 0.001 to 0.117) genomic differentiation between sampling locations based on the putatively adaptive dataset, with the majority significant and higher than observed with the putatively neutral dataset [29] (see Additional file 1: Fig. S5; Table S4). The greater F_ST_ differentiation was between GSV and EWP compared to the western sites [ALB and ESP], with the lowest F_ST_ values showcasing a gradient along southern Australian sites on the continental shelf.

### Functional enrichment and annotation

**Fig. 5:**
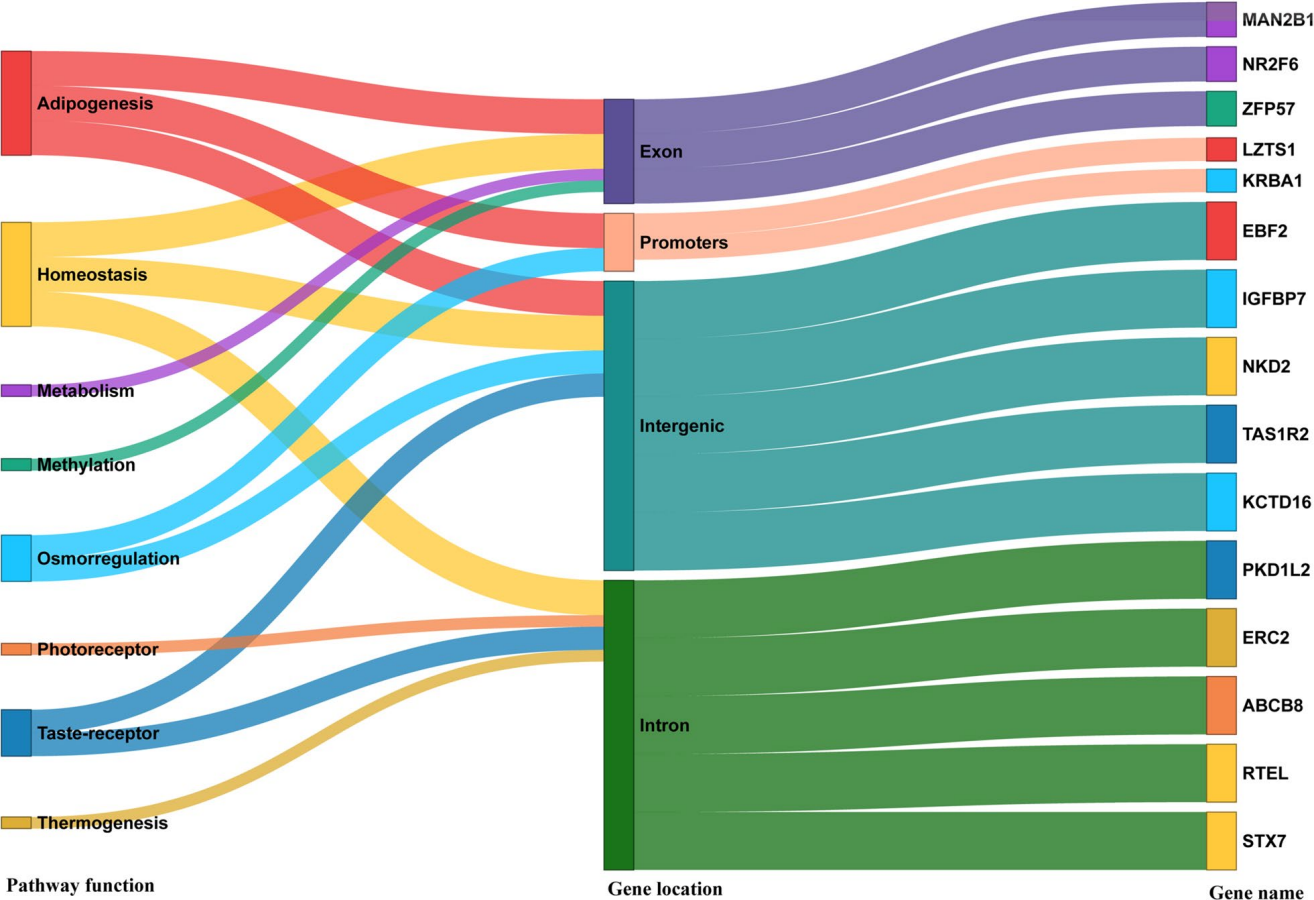
Sankey diagram showing the 15 of the 26 candidate genes disclosed by the functional enrichment analysis, their interlinkage pathways, and gene location from this study. Colours of each gene corresponds to the function pathway and colours of gene location are only displayed for visual representation

A total of 1871 SNPs, from the full dataset of 17,327 SNPs (> 10%), scored BLAST hits to the publicly available cetacean nucleotide and non-redundant protein databases (NCBI). Of the 747 potentially adaptive SNPs, 148 were annotated (~ 19%). Functional enrichment analysis identified 22 GO terms over-represented in the putative adaptive loci (Additional file 1: Table S5). These over-represented GO terms belong mostly to biological processes, with 26 adaptive candidate SNPs in 15 known genes and associated to multiple pathways (Fig. 5).

These 26 SNPs were further investigated, with three of them occurring in exonic regions (Figs. 5; Additional file 1: Fig. S6; Table S6). These SNPs corresponded to candidate genes MAN2B1, which is related to breaking complex sugar molecules in carbohydrate metabolism [57–59], and ZFP57 which is related to early embryonic methylation that is potentially altered by nutrients in one’s diet [60–62]. These SNPs were most strongly associated with primary productivity variation in the RDA. The third SNP in an exonic region correspond to candidate gene NR2F6 (also known as CoupTFII), which is related to the regulation of adipogenesis, glucose, homeostasis and energy metabolism [63–65], and was correlated with the salinity gradient in the RDA. Although the correlations between candidate SNPs and the environmental variables were generally low (r^2^ < 0.5), the correlations were still significant (Fisher’s p-value < 0.05), and considerable variation in allele frequencies of these candidate SNPs were observed across the seascape (Additional file 1: Fig. S7). Other candidate SNPs were located in promoters (LZTS1, KRBA1), intronic regions (PKD1L2, P3H2, SYT6, ERC2, LEF1, ABCB8, RTEL1, TLN2, MTCL1, STX7 and CFAP54), and intergenic parts of putative genes (LZTS1, EBF2, ELL, IGFBP7, KRBA1, NKD2, TAS1R2, MTCL1 and KCTD16).

## Discussion

Species and populations exhibiting high genomic variation have enhanced prospects for long-term persistence [66–68]. However, highly diverse populations inhabiting multiple environments may be subject to disparate selective pressures, which in turn can result in adaptation to particular habitats [2, 3, 69]. Marine predators are expected to be highly impacted by complex and indirect ecological interactions between their prey and habitats (e.g., [70]). For high dispersal marine species, such as common dolphins, little is known about the spatial distribution of adaptive diversity and its association with spatial connectivity and population subdivision. Here, a seascape genomics approach was used to assess the influence of environmental heterogeneity in shaping putatively adaptive divergence in common dolphins from southern Australia. Our analyses identified over 700 SNPs putatively under selection that delineated up to five populations across the region. The seascape genomics approach revealed four key environmental variables (sea surface temperature, primary productivity, current velocity, and salinity) influencing patterns of genomic variation. This genomic signal appears associated with three different oceanographic phenomena in southern Australian coastal and shelf waters: (1) oceanographic circulation patterns in the western region and related differences in current velocity; (2) upwelling hotspots across the central shelf region influencing fluctuations of primary productivity and sea surface temperatures; and (3) protected coastal environments in the central and eastern bioregions characterised by marked variations in salinity and seasonal circulation patterns.

### Candidate adaptive genomic variation in southern Australian common dolphins

Genomic variation within populations is impacted by demographic history, but also through ongoing selective pressures that will promote or restrict the dispersal of individuals, which may in turn be reinforced by social structure and behaviour [71, 72]. The comparison between candidate adaptive markers (this study) and previously reported neutral markers [29], showed that estimates of genomic variation and differentiation were higher for the adaptive markers. More complex population structure was also revealed, with five supported populations suggested here using the candidate adaptive markers, compared with just two supported for the neutral dataset [29]. While neutral and adaptive genetic signals both provide useful information about population structure, they arise through different evolutionary forces [68, 73], and can inform different aspects of conservation and management requirements [74–76], as discussed below.

Understanding the pressures that impact marine populations is critical for guiding their management [55]. Common dolphins are a widespread species thought to have colonised different coasts and pelagic habitats off Australia during the Pleistocene [77]. During that period, fluctuations in primary productivity may have opened new niches and promoted colonisation of the region by this species [78–80]. However, contemporary environmental pressures may also influence local adaptation of marine populations [66, 81]. This is relevant for common dolphins in southern Australia, which experience ongoing impacts from anthropogenic activities, particularly by-catch in multiple fisheries (e.g., [38, 82, 83]). Common dolphins exhibit two patterns relevant for their management and conservation. From a neutral perspective, based on signals from genomic regions mostly shaped by genetic drift and migration, genetic connectivity is observed over relatively long geographical distances [29]. From an adaptive perspective, signatures of putative selection supporting local adaptation to some embayment environments, which corroborates field observations of site fidelity and year-round residency in these areas (e.g., [44, 84, 85]). Neutral and adaptive loci can disclose a different number of populations for management and conservation [76, 86]. For common dolphins in southern Australia, adaptive loci supported the hypothesis of putative adaptation to local environments or bioregions that may provide long-term evolutionary potential despite the occurrence of gene flow at the metapopulation level, as previously found with neutral loci [29]. Each local habitat, or bioregion, exhibits different environmental gradients, such as temperature, current velocity, and salinity, which could be impacting common dolphins and their prey distribution in southern Australia. Differentiation between locally adapted populations despite metapopulation gene flow, has been previously described for other marine taxa along southern Australia (e.g., *Haliotis* [36, 37], and *Nerita* [87]), and in another delphinid (e.g., *T. aduncus* [20]). Effects of climate change may differ between adapted populations, with local populations exhibiting low adaptive diversity, such as those in coastal protected environments or embayments, perhaps more vulnerable to such effects (e.g., [20, 40, 88]). Thus, neutral and adaptive variation are both relevant to conservation for maintaining high standing genetic variation and evolutionary potential across the southern Australia’s common dolphin metapopulation.

### Environmental drivers of adaptive differentiation in southern Australian common dolphins

Adaptive differentiation can be driven by various selective pressures. In marine systems, oceanographic features, such as bathymetry, currents, primary productivity, salinity, and temperature may exert selective pressures [4, 55, 89]. Environmental gradients and discontinuities could be creating soft barriers, and in turn, lead to adaptive divergence among marine populations or bioregions (e.g., [4, 36, 90]). Population differentiation in southern Australian common dolphins has been generally associated with their social structure, areas of high primary productivity, as well as abundance and movements of their prey (e.g., [31, 44, 85]), which mainly exhibit passive dispersal during their larvae stage (e.g., [91–93]). Although using a small representation of the genome in the current study led to a significant, albeit small, association between genomic variation with key environmental variables, it does not necessarily fully elucidate the complex scenarios that occur in the marine system. The assessment of common dolphin populations in southern Australia, thus requires a general ecological understanding of this heterogeneous marine system.

At a global scale, a seascape genetics study of common dolphins based on neutral microsatellite markers found evidence for chlorophyll *a* and sea surface temperature variation linked to major population boundaries [34]. Robust analyses in our study based on a large SNP dataset disclosed that common dolphin genomic variation was associated not just with sea surface temperature, but also current velocity, primary productivity (which is generally positively correlated with chlorophyll *a*), and salinity. Our findings suggest that different environmental variables may be acting on common dolphins’ genomic variation in different bioregions, playing major roles in the differentiation of their populations. The ocean circulation in the region, which influences seasonal differences in primary productivity and sea surface temperature, likely results in different levels of plankton biomass (e.g., [52]), and in turn mediates the abundance and distribution of the dolphins’ prey.

#### Ocean circulation off southern Western Australia

The western and southern coasts of Western Australia are characterised by the Leeuwin current, which originates in the warm Indo-Pacific Ocean, moving south along the western coast of Australia, and into the southern coast off Cape Leeuwin [94–96]. The warm waters that enter southern Australia, flowing from west to east, create distinct patterns of temperature, primary productivity, and current velocity along the continental shelf [97, 98]. In southern Western Australia, common dolphins from the two geographically close sites of Albany and Esperance (~ 300 km apart) were found to be differentiated based on the adaptive SNP dataset, and this distinction seems to be mainly driven by oceanographic currents, particularly changes in current velocity in the region (Figs. 2c, d; 3). This result is consistent with a previous microsatellite study that suggested differentiation between dolphins of these two sites [31], although this separation was not disclosed by neutral SNP study [29]. A recent GEA study in a closely related species, the Indo-Pacific bottlenose dolphin (*T. aduncus),* did not disclose similar separation between localities in the southern coast of Western Australia [20]. This is potentially due to difference in habitat use between the two species, with bottlenose being generally found closer to shore as compared to common dolphins that have a more offshore distribution.

In the western region of southern Australia, the Leeuwin current velocity rapidly declines due to geomorphological formations at the ocean floor, such as the presence of canyons and the Recherche archipelago [93, 96, 99]. Models of larval dispersal in some fish species have shown that individuals move along the Leeuwin current, but separate fish aggregations form between Albany and Esperance [100]. Circulation and geomorphological differences characterise each site; Albany with strong mixing of waters outside the embayment, whereas off Esperance waters are more protected because of the archipelago’s presence [101, 102]. Common dolphins are known to optimise their energy requirements by having different hunting strategies and preferences for targeting diverse prey species between different sites and seasons [30, 103, 104]. Thus, common dolphins that inhabit Albany and Esperance could be targeting different fish aggregations, which may lead to differential habitat use.

Interpretation of functional implications between SNPs and their environment needs to be made with caution, especially in marine systems as it is difficult to elucidate all possible oceanographic and demographic scenarios [105, 106]. However, adaptive genomic variation of common dolphins between Albany and eastern sites was evident in SNPs of the candidate genes EBF2, LEF1 and KCTD16. Variation at these genes correlated with current velocity and primary productivity. These genes are known to be linked to pathways of adipogenesis, homeostasis, thermogenesis and osmoregulation, which promote digestion, absorption of carbohydrates, hypoxic conditions, energy conversion, as well as differentiation of brown adipocytes (e.g., [107–109]).

#### Southern Australia’s continental shelf and its upwellings

The Leeuwin current continues into southern Australia as the Great Australian Bight current, which is characterised by slower flow in an eastward direction following the break of the continental shelf [95, 97, 110]. The sea floor formation of the southern continental shelf of Australia creates a basin known as the Great Australian Bight, which extends several nautical miles from the coast into the continental shelf break [111–113]. While the warm currents tend to follow the continental shelf, there is also a counter-current of cold water, known as the Flinders current, which typically remains off the continental shelf break [97]. During the austral summer, anticyclonic weather favours the replacement of the warm currents by the cold and productive Flinders current, forming coastal upwellings over the continental shelf [46, 47, 95]. The GEA analysis highlighted the influence of these upwellings as demonstrated by the importance of maximum primary productivity and minimum sea surface temperature in shaping the adaptive genomic differentiation of common dolphins. This was particularly reflected in samples from regions of the Great Australian Bight, mouth of Spencer Gulf, Robe, Portland, and Melbourne, which clustered together, a pattern also disclosed in the PCA and Admixture analyses.

Differences in primary productivity and sea surface temperatures have been considered the main forces that drive seasonal upwellings in southern Australia [46, 100, 112]. There are two types of upwelling centres along southern Australia. Large upwellings are represented by the Bonney upwelling located between Robe, South Australia, and Portland, Victoria, and the Tasmanian upwelling located off western Tasmania. There are also smaller upwelling centres that do not follow the classical Eckman model formation, such as the Eyre Peninsula and the Kangaroo Island upwellings [99, 110, 112]. While the smaller upwellings could mainly have an impact upon the spawning of fish species, such as sardines and anchovies [91], the larger upwelling centres attract a high density of predators, such as dolphins, whales, seals and sharks feeding upon large biomasses of krill and pelagic fish (e.g., [114–116]).

Common dolphins in open and unprotected continental shelf waters (i.e. Great Australian Bight, mouth of Spencer Gulf, Robe, Portland and Melbourne) presented similar Minor Allele Frequency (MAFs) in the SNPs of candidate genes ERC2 (intronic), and MAN2B1 and ZFP57 (exonic) (SNPs correlated to either primary productivity or temperature), compared to other localities (Additional file 1: Figs. S6 and S7). In other taxa, these genes have been associated with heat stress metabolism, the breaking of complex sugar molecules, and the regulation of fatty acids [59, 117, 118].

#### Protected coastal habitats

The geomorphology of southern Australia includes several embayment and protected areas [94, 96]. Some of these protected embayments, such as Gulf St Vincent in South Australia, and Port Philip Bay in Victoria, have been previously described as important year-round habitats for common dolphins [44, 84, 103]. A previous study using a neutral genomic dataset suggested some differentiation between dolphins from Gulf of St Vincent and other areas [29]. In our study, this differentiation was further informed by the GEA analysis, which disclosed that the genomic variation of common dolphins in sheltered waters of Gulf St Vincent and East Wilsons Promontory is apparently driven by variations in salinity, primary productivity and sea surface temperatures. The Gulf St Vincent is a hyper-saline inverse estuary (i.e., salinity increases with distance to the mouth, and evaporation exceeds inflow circulation), with seasonal circulations that create differences in primary productivity and temperatures [96, 119, 120]. Similarly, Wilsons Promontory is a protected area described as a unique biogeographic region between the southern and eastern Australian currents, with seasonal circulation associated with differences in temperature that promotes the formation of seasonal fish assemblages [49, 121, 122].

In southern Australia, common dolphins mainly feed upon sardines (*S. sagax*), anchovies (*E. australis*), and mackerel (*Trachurus* spp*.*) [33, 123], which are prey species with high energy density. However, for some presumably resident populations in Port Phillip Bay and Hauraki Gulf, New Zealand, it has been suggested that common dolphins may change their target species based on seasonal availability [30, 103]. Bottlenose dolphins that exhibit strong residency in embayment habitats in southern Australia have also shown genomic differentiation associated with salinity, current velocity and temperature (see Pratt et al. [20] for details), variables than influence temporal fish composition in the region (e.g., [119, 120]), and could create indirect discontinuities in food availability [20]. For bottlenose dolphins, it was hypothesised, based on genes found to be under potential selection, that some physiological adaptations could be occurring at a population level, especially in embayment habitats [20]. For southern Australian common dolphins from coastal protected habitats, especially those from Gulf of St Vincent, there were significant genomic differences by RDA and enrichment analysis in the MAF of SNPs in genes STX7 and IGFBP7 (correlated to salinity), which are genes involved in pathways of osmoregulation and other physiological adaptations (e.g., [124–126]). Macroevolutionary studies of odontocetes have suggested that adaptative divergence mostly occurred during cycles of high productivity [78–80]. This was observed, for example, in members of the STX family genes which are positively selected in the macroevolution of marine mammals (e.g., [16, 127, 128]).

In southern Australian embayments, seasonal changes in salinity and temperature are associated with changes in the composition of fish assemblages (e.g., [122, 129]). Genomic variation in common dolphins inhabiting embayment areas, such as Gulf St Vincent, could relate to mechanisms for coping with high salinities, while allowing them to remain locally resident year-round by alternating feeding upon different prey species. Although the evidence reported here is based on a small representation of the genome (less than 2% of the dolphin genome with a 99% alignment to *T. aduncus*), it is expected that future studies using species-specific whole genomes, will expand and report on many other gene regions and pathways likely to be under selection in these common dolphin populations.

### Implications for conservation under future climatic scenarios

This study points to environmental variables that may be influencing putatively adaptive populations of common dolphins across southern Australia. With rapid and ongoing climatic change and other anthropogenic pressures impacting on marine species [3, 11, 130], it is essential to understand which environmental factors shape genomic variation to identify locally adapted populations relevant for conservation and management. Models predicting the impact of climate change in marine systems have provided evidence that differences in circulation patterns will likely lead to warmer environments [131–133]. For cetaceans, two possible scenarios have been proposed. One scenario suggests that changes in ocean circulation, wind patterns and currents could enhance upwelling areas, with large predators such as common dolphins likely benefiting from these changes (e.g., [41, 134, 135]). In contrast, another scenario suggests that warmer sea surface temperatures would alter community dynamics and increase exposure of populations to various pathogens (e.g., [40–42]). In this second scenario, prey abundance and distribution could be greatly impacted by effects on plankton biomasses (e.g., [136, 137]), which could lead to disease outbreaks, prey depletion and population declines for cetaceans and other marine predators.

Potential effects suggested in both scenarios could impact southern Australian common dolphins. The first scenario is perhaps most likely for common dolphin populations inhabiting sites where connectivity persists over thousands of kilometres due to seasonal aggregations in the upwelling areas (e.g., [41, 135]). Southern Australian common dolphins could enhance the movement of nutrients to different habitats and trophic levels (e.g., [138, 139]). This movement of nutrients due to climate change could affect the timing and magnitude of the upwellings, which could deplete some areas and eutrophicate others (e.g., [136]), impacting common dolphins and other cetacean species that feed upon high density prey biomasses [137, 139, 140]. In contrast, the second scenario could potentially be more relevant for common dolphins that live in protected habitats in which extreme climatic events, such as marine heatwaves, could lead to high mortalities of prey species (e.g., [141]) and alteration of spawning times (e.g., [92, 142]). Moreover, changes in temperature and nutrients of waters masses could lead to low abundance and redistribution of prey species (e.g., [143, 144]). For dolphin species that inhabit protected environments, epizootic events often coincide with these types of extreme climatic stressors, leading to negative impacts on population health and reproduction, and occasionally large morbidity and mortality events (e.g., [40, 43, 145]). Moreover, common dolphins in embayment areas such as Gulf St Vincent and Spencer Gulf have been subjected to fisheries interactions for long periods of time [28, 38, 56], and extreme climatic events leading to lower food availability may further compound negative impacts (e.g., [40, 43, 140]). The dynamics of marine ecosystems are extremely complex, and future climatic changes may lead to indirect effects that need to be contemplated in future conservation planning [46, 144].

As a near top predator, common dolphins provide important ecosystem services to the marine environment [138, 139], and if further anthropogenic or climatic impacts were to occur, these could lead to changes in food-webs potentially causing the eutrophication of ecosystems (e.g., [40, 146, 147]). Adaptation to heterogeneous environments in species with high genomic diversity can promote population resilience to climatic changes [72]. When developing policies and management decisions, it is important to incorporate information from both neutral and adaptive markers to ensure the persistence of high standing genomic variation in marine populations [2, 69, 148]. Currently, these putatively divergent dolphin populations are being managed according to the management stocks of their prey (e.g., [149]), with no specific consideration of common dolphin genetic or genomic differentiation. Results of this study disclosed five putatively adaptive common dolphin populations in southern Australia that need to be considered as priority areas for conservation and management, taking into account the potential cumulative impacts of fisheries (e.g., [38]) and other stressors on each local population, as well as across its Australasian metapopulations [29]. In line with Funk et al.’s [86] framework for the delineation of conservation units, we suggest that common dolphin management units should incorporate results of both neutral [29] and adaptive population structure. Integrating these results can lead to management units that enhance functional corridors, and provide long-term, high standing genetic variation to the populations. We also recommend that future common dolphin studies should implement this framework as already used for other marine taxa (e.g., *Parastichopus* [12], *Microtus* [150], *Carcharhinus* [151]). Management units from areas such as Gulf of St Vincent should be prioritised, given it continues to be impacted by human activities, exhibits the least amount of migration compared to adjacent sites [29], and is an area where common dolphins show putative adaptation to the semi-enclosed embayment.

## Conclusion

Our study suggest that conservation and policy efforts towards common dolphins should preserve diversity as well as connectivity, and take into consideration cumulative impacts on the putatively adaptive populations as a proxy of evolutionary potential. The GEA analysis indicated that common dolphin genomic variation is impacted by four key environmental variables, which in turn are likely related to three oceanographic phenomena that characterise this broad ocean region. Genomic variation in dolphins off the southern coast of Western Australia was associated with current velocity, while genomic differentiation of common dolphins from sites along the continental shelf break were associated with primary productivity and sea surface temperature. The latter may relate to major upwelling centres, which could be promoting areas of seasonal aggregation. In contrast, genomic differentiation of common dolphins from protected coastal habitats and embayments were associated mainly with fluctuations in salinity. These environmental variables present gradients and discontinuities, which may create soft barriers among the putative populations. Thus, it is recommended that neutral and adaptive variation should be considered for the management of these five putatively locally adaptive common dolphin populations, while allowing long-range gene flow to persist across their previously described Australasian metapopulation. Maintaining connectivity can promote long-term high standing genomic variation, which in turn will enhance population viability under anthropogenic impacts, including unfavourable climatic events. Furthermore, this study represents the first seascape genomic assessment of common dolphins in a dynamic heterogeneous environment. The candidate genes described here may be useful for future comparative studies of common dolphins and potentially other delphinid species that share similar environments.

## Methods

### Sample collection and study area

Common dolphins were sampled across > 3000 km of southern Australian waters between 2002 and 2015, with locations allocated based on individual GPS data (Fig. 1). These sites encompass the distribution of this species along the oceanographic, environmental, and geological discontinuities of southern Australia. Biopsy samples were collected from live individuals using a hand held biopsy pole [152] or remote biopsy gun (PAXARMS) [153]. Dependent calves were not sampled to avoid the inclusion of closely related individuals. Biopsy samples were preserved in 90% ethanol or in a 20% salt-saturated solution of dimethyl sulphoxide (DMSO) and stored frozen (− 80 °C) until laboratory analyses took place. A total of 234 biopsy samples were used for the genomic analyses.

### Laboratory analyses and bioinformatics

DNA extractions from biopsy samples were performed using a salting-out protocol [154]. Quantity and quality controls of DNA were determined using a Qubit 2.0 fluorometer (Life 178 Technologies), a Nanodrop spectrophotometer (Thermo Scientific), and gel electrophoresis. Library preparation for the double digest restriction-site associated (ddRAD) was performed following the protocol of Peterson et al. [155], and sequencing was done using an Illumina HiSeq 2500 platform producing single-end, 100 bp reads, at the South Australian Health & Medical Research Institute (SAHMRI).

Raw data quality was assessed, and demultiplexed using process_radtags, with STACKS v1.48 [156, 157], reads were trimmed by quality (only one error allowed) using TRIMMOMATIC [158], and then dDocent2.2.19 pipeline [159] was used to call the SNPs. The resulting loci were then filtered using VCFtools (see details in Additional file 1 and [29]). The quality-filtered ddRAD loci were then mapped against the genome of a closely related species, the Indo-Pacific bottlenose dolphin (*T. aduncus*) from southern Australia [145] using Bowtie2 [160], due to the absence of a high-quality *D. delphis* genome*.* Genotype errors were considered and filtered by the incorporation of 10 known replicate samples. To exclude potential field sample duplicates or closely related individuals in the dataset, relatedness between pairs of individuals was calculated by the triadic likelihood estimator (TrioML) in Coancestry v1.0.1.9, as this estimator provides the highest correlation with true values [161]. Then, following a simulation protocol of Attard et al. (see [162] for details of parameters), we determined a relatedness threshold (|R|> 0.5) to remove one individual per each pair of duplicates or closely related individuals, such as parent-offspring (detailed in Additional file 1: Table S1).

### Selection of environmental variables and spatial data

Bathymetry, sea surface temperature, chlorophyll *a*, current velocity, primary productivity, and salinity were selected as ecologically relevant environmental variables to test for associations with common dolphin genomic variation based on previous studies (e.g., [29, 31]). For each of the variables selected, the annual maximum, mean, minimum, and range values between the years 2000 and 2014 were used, resulting in a total of 24 variables (Additional file 1: Table S2). The environmental data was downloaded from the database BioOracle at a resolution of ~ 9.2 km, using the R package ‘sdmpredictors’ [163, 164] (see Additional file 1 for details).

To control for spatial autocorrelation, pairwise oceanic distances were calculated using the GPS coordinates of each individual and the R function *viamaris* available at https://github.com/pygmyperch/melfuR. Pairwise oceanic distances were then transformed to Moran’s eigenvector maps (MEM) using the package ‘memgene’ with a forward selection procedure, 100 permutations, and an alpha value of 0.05 [165]. The selected MEMs were then used as the spatial variables for analyses.

To determine which environmental variables were significantly driving the population genomic differentiation from the initial 24 environmental variables, standardisation was first implemented with the R package ‘pysch’ [166], before excluding highly correlated variables (*r* > 0.7) [167–169] and those with a variance inflation factor (VIF) ≥ 3 [170]. We then used a forward selection criteria with the R package ‘vegan’ [171] to retain environmental variables that explained a significant (p < 0.05) portion of the genomic variation [166, 172].

### Genotype-environment association

Loci putatively under selection were detected using a seascape genomics approach within an individual-based genotype-environment association (GEA) framework. This methodology allows the identification of associations between genetic and selected environmental variables across individuals, with multivariate analyses carried out in the R package ‘vegan’ [171] (see description and parameters of analysis below). The Redundancy Canonical Analysis (RDA) was chosen because it usually outperforms other GEA methodologies, such as univariate analyses (e.g., Latent Factor Mixed Models), for detecting genomic markers associated with environmental variables, as it reduces the number of false-positives without compromising the detection of true-positive candidates (e.g., [173, 174]). A partial RDA was used to assess the effect of the selected environmental variables on the genomic diversity while controlling for the spatial pattern using the selected MEMs.

The significance of each environmental variable and axis (p < 0.05) was calculated using an Analysis of Variance (ANOVA) with 1000 permutations. Loci that scored greater than three standard deviations (± 3SD) from the mean locus scores were selected as candidates for each of the significant RDA axes. Selected axes explained a significant (p < 0.05) portion of the genomic variation, as previously suggested [172, 173]. Spearman’s correlations were then calculated between each of the candidate loci and the retained environmental variables to determine the most important environmental variable shaping allele frequencies of each locus.

### Adaptive population diversity and structure

Genomic diversity of the candidate SNPs was assessed for each sampled site using GenoDive 2.0b27 [175]. Principal Component Analysis (PCA), which is a model-free approach, was used to investigate population structure using the R package ‘Adegenet’ [176, 177]. The Akaike Information Criterion (AIC) was then used to determine the best-supported number of clusters in the dataset, using the snapclust.chooseK function, also in ‘Adegenet’ [178].

Population structure was further investigated using a Bayesian clustering approach that infers population stratification based on the estimated individual ancestries in Admixture v1.3.0 [179]. Although the candidate adaptive dataset likely violates Hardy–Weinberg assumptions of equilibrium [86], this analysis was used as a comparison to the results based on the putatively neutral dataset [29]. The maximum likelihood estimates were calculated by using the ancestry portion and the population allele frequency to assign the most likely number of K (i.e., populations) in the dataset, testing for K 1–9, and for modelling the probability of observed genotypes [180]. This was followed by cross validation with ten replicates for each of the K values.

Adaptive genomic differentiation among sites was estimated as pairwise F_ST_ [181] using GenoDive 2.0b27. Significance levels were assessed using 10,000 permutations, corrected by the B-Y method (FDR < 10%; [182]). Heatmap plots of F_ST_ were constructed with the R package ‘ggplot2’ [183].

### Functional enrichment analysis and annotation

A functional enrichment analysis was performed for the full annotated dataset of 17,327 SNPs as a background gene set, using the nucleotide and non-redundant protein NCBI databases for all available cetacean sequences [53, 184–186]. Based on the results of linkage disequilibrium (details in [29]), flanking sequences of 300 bp either side of each SNP were extracted, resulting in 601 bp sequences. Annotation was then performed using the alignment tool (BLAST) in the NCBI nucleotide and non-redundant protein databases, with an e-value threshold of 1 × 10^−3^. A Gene Ontology (GO) term enrichment analysis was also performed comparing the candidate loci to the full dataset (17,327 SNPs) using a Fisher’s exact test and a FDR cut-off of ≤ 5% [187]. The resulting GO terms related to a specific SNP in a candidate gene, were then further examined for its sequence position using snpEFF [188]. Each SNP was further assigned to a pathway and function using the Reactome [189] and UnitProtKB databases, respectively (The UnitProt Consortium, 2018).

## Supplementary Information

**Additional file 1: Table S1.** Filtering steps and number of SNPs retained after each step for common dolphins (*Delphinus delphis*) in southern Australia. FDR: false discovery rate. **Table S2.** Summary of environmental variables included in the genotype-environment association multivariable analyses, datasets were retrieved from BioOracle. **Table S3.** (**A**) Significance of the RDA and the proportion explained by each component of the full model selection. (**B**) Significance of the environmental variables selected, with an overall significance of the model and variables at *p* = 0.0001. **Table S4.** Pairwise F_ST_ values between sites based on putatively adaptive (this study) and neutral (1) datasets for southern Australian common dolphins (*Delphinus delphis*). Upper right, neutral F_ST_ values, and lower left adaptive F_ST_ values, with their significance of the p-values by the B-Y method corrected represented by ***0.0001, **0.001. Acronyms for sites as in Fig. 1. **Table S5.** Significance of Gene Ontology (GO) terms for southern Australian common dolphins (*Delphinus delphis*), comparing the full dataset with the putative candidate loci by Fisher’s exact test. Biological Process (BP), Molecular Function (MF), Cellular Component (CC). **Table S6.** Function of the candidate genes found in exonic regions, which were over enriched by the Gene Ontology analyses, for the 747 putatively adaptive SNPs discovered by the RDA of southern Australian common dolphins (*Delphinus delphis*). **Figure S1.** Multicollinearity between the five environmental variables used for the RDA. Salinity maximum (BO2_salinitymax_ss), primary productivity maximum (BO2_ppmax_ss), sea surface temperature minimum (BO_sstmin), current velocity maximum (BO2_curvelmax_ss) and current velocity range (BO2_curvelrange_ss). Numbers in the upper right matrix are the correlation values of each comparison; the smaller the number gets, the closest to zero the correlation between the variables is. Variables were standardised from 0-1. **Figure S2.** Akaike Information Criterion (AIC) used to determine the best-supported number of clusters in the PCA, based on the putative adaptive dataset of southern Australian common dolphins (*Delphinus delphis*). **Figure S3.** Population genomic structure analysis using Admixture based on the putatively adaptive SNPs for southern Australian common dolphins (*Delphinus delphis*) (labelled by sampling site and individual). K represents the number of populations tested (**A** to **F**), in which K4* and K5* are both correctly assigned as they are the most supported and highly likely number of local populations suggested by the analyses. Acronyms for sites as in Fig. 1. **Figure S4.** Principal Component Analysis (PCA) based on 747 candidate adaptive loci for southern Australian common dolphins (Delphinus delphis). (**A**) Explanatory axes PC1 vs PC2. (**B**) Explanatory axes PC1 vs PC3. (**C**) Explanatory axes PC2 vs PC3. Acronyms for sites as in Fig. 1. **Figure S5.** Heatmap of pairwise F_S__T_ values between sites based on the adaptive (this study) and neutral (1) SNP datasets for southern Australian common dolphins (*Delphinus delphis*). Upper right, neutral dataset, and lower left, adaptive dataset. Acronyms for sites as in Fig. 1. **Figure S6.** Allele frequency changes for the candidate gene variants found in exonic regions of common dolphins (*Delphinus delphis*) across sampling sites in southern Australia. (**A**) NR2F6/NR2F2 was associated with maximum salinity, (**B**) ZFP57, with primary productivity maximum, and (**C**) MAN2B1, with primary productivity maximum. **Figure S7.** Minor allele frequencies of 26 SNPs selected by a gene enrichment test, with variants in coding or non-coding regions.

## Data Availability

The datasets generated and/or analysed during the current study are available in the FigShare repository https://figshare.com/s/f6e85619698e8a90b8f3 (https://doi.org/10.6084/m9.figshare.19319732).
