# Supplementary material for: Seascape genomics of common dolphins (Delphinus delphis) reveals adaptive diversity linked to regional and local oceanography
Source: BMC Ecol Evol. 2022 Jul 12;22:88. doi: 10.1186/s12862-022-02038-1 (PMC9275043; doi:10.1186/s12862-022-02038-1)
Supplement: Supplementary file 1 — Additional file 1: Table S1. Filtering steps and number of SNPs retained after each step for common dolphins (Delphinus delphis) in southern Australia. FDR: false discovery rate. Table S2. Summary of environmental variables included in the genotype-environment association multivariable analyses, datasets were retrieved from BioOracle. Table S3. (A) Significance of the RDA and the proportion explained by each component of the full model selection. (B) Significance of the environmental variables selected, with an overall significance of the model and variables at p = 0.0001. Table S4. Pairwise FST values between sites based on putatively adaptive (this study) and neutral (1) datasets for southern Australian common dolphins (Delphinus delphis). Upper right, neutral FST values, and lower left adaptive FST values, with their significance of the p-values by the B-Y method corrected represented by ***0.0001, **0.001. Acronyms for sites as in Fig. 1. Table S5. Significance of Gene Ontology (GO) terms for southern Australian common dolphins (Delphinus delphis), comparing the full dataset with the putative candidate loci by Fisher’s exact test. Biological Process (BP), Molecular Function (MF), Cellular Component (CC). Table S6. Function of the candidate genes found in exonic regions, which were over enriched by the Gene Ontology analyses, for the 747 putatively adaptive SNPs discovered by the RDA of southern Australian common dolphins (Delphinus delphis). Figure S1. Multicollinearity between the five environmental variables used for the RDA. Salinity maximum (BO2_salinitymax_ss), primary productivity maximum (BO2_ppmax_ss), sea surface temperature minimum (BO_sstmin), current velocity maximum (BO2_curvelmax_ss) and current velocity range (BO2_curvelrange_ss). Numbers in the upper right matrix are the correlation values of each comparison; the smaller the number gets, the closest to zero the correlation between the variables is. Variables were standardised from 0-1. Figure S2. [file 12862_2022_2038_MOESM1_ESM.docx]

# Supplementary Material: Seascape genomics of common dolphins (*Delphinus delphis*) reveals adaptive diversity linked to regional and local oceanography

Common dolphin (*Delphinus delphis*) samples used for this study cover nine sites along the southern Australian coastline (>3,000 km), resulting in a panel of 17,327 SNPs based on 234 samples (Table S1). Samples are analogous to a previous study of putatively neutral genomic markers that aimed to clarify population structure and gene flow of common dolphins at different scales (1). Using the aligned reads from (1), the data was filtered based on the parameters listed in Table S1, Step 10. The 234 common dolphin samples from southern Australia were then subsampled from the Australasia dataset. Closely-related individuals were removed and then filtered for a minor allele count (MAC<3; Step 11). This last filter was applied to remove singletons, which can inflate genetic differentiation between populations with high connectivity (2). This resulted in a final dataset of 17,327 SNPs and 214 individuals. For this study, geographical distances between different environments were taken into account and samples were separated into the nine sampling sites, which provided a similar number of samples per site to compare the influence of environmental variables on the genomic variation of common dolphins.

Environmental variables used for the genotype-environment association (GEA) multivariable analyses were downloaded from the latest BioOracle datasets using the R package ‘sdmpredictors’ (3, 4) (Table S2). Selected variables from BioOracle were originally measures from the Moderate Resolution Imaging Spectroradiometer (Aqua-MODIS) and *in situ* measurements, corresponding to sea surface temperature, salinity, primary productivity, current velocity and chlorophyll *a* (3). These variables were then reanalysed by Global Ocean Physics Non-assimilative Hindcast (PISCES) and Global Observed Ocean Physics Reprocessing (ARMOR) for a monthly average measure from 2000-2014 and scaled to a resolution of ~9.2 km (4). Bathymetry is based on measures from the General Bathymetric Chart of the Oceans (GEBCO) (3, 4). The range of bathymetry was not available from the BioOracle database; therefore, it was calculated from the recoded GPS location of each individual sample, based on the range of the minimum and the maximum values of the bathymetry.

**Table S1.** Filtering steps and number of SNPs retained after each step for common dolphins (*Delphinus delphis*) in southern Australia. FDR: false discovery rate.

| **Filtering Step** | **SNP count** |
| --- | --- |
| **1. Raw SNP catalogue after STACKs, TRIMMOMATIC and dDocent pipeline** | 339,932 |
| **2. Genotyped in 80% of individuals, base quality ≥ 30, minor allele frequency >0.03 and bi-allelic (using VCFtools)** | 33,467 |
| **3. Split multiple nucleotide polymorphisms into SNPs (using VCFtools)** | 33,010 |
| **4. Read depth ≤ mean depth + (2 x standard deviation) (using VCFtools)** | 32,031 |
| **5. Read quality (ratio quality/coverage depth > 0.2) (using VCFtools)** | 31,160 |
| **6. Allele balance > 20% and < 80% (using VCFtools)** | 31,020 |
| **7. Hardy–Weinberg equilibrium in > 80% localities (using VCFtools)** | 29,224 |
| **8. Present in at least 75% of individuals and in each putative population (using VCFtools)** | 26,431 |
| **9. Alignment against southern Australian bottlenose dolphin, *Tursiops aduncus* genome (Batley et al., 2021) (using Bowtie2)** | 26,199  (99% alignment) |
| **10. Best quality SNP (higher average Q) within 330bp. Sub-set of 234 subsamples for southern Australian common dolphins from the Australasian dataset (using VCFtools)** | 17,875 |
| **11. Minor allele count <3 based on the dataset excluding closely-related individuals (\|R\|** >**0.5), i.e. 214 samples for southern Australian common dolphins (using VCFtools)** | 17,327 |
| **Average missing data per locus** | 1% |

**Table S2**. Summary of environmental variables included in the genotype-environment association multivariable analyses, datasets were retrieved from BioOracle.

| **Name** | **Layer code** | **Units** | **Resolution** | **Date** |
| --- | --- | --- | --- | --- |
| Sea surface temperature (maximum) | BO_sstmax | Celsius | ~9.2km | 2000-2014 |
| Sea surface temperature (mean) | BO_sstmean | Celsius | ~9.2km | 2000-2014 |
| Sea surface temperature (minimum) | BO_sstmin | Celsius | ~9.2km | 2000-2014 |
| Sea surface temperature (range) | BO_sstrange | Celsius | ~9.2km | 2000-2014 |
| Chlorophyll concentration (maximum) | BO2_chlomax_ss | mg/mü | ~9.2km | 2000-2014 |
| Chlorophyll concentration (mean) | BO2_chlomean_ss | mg/mü | ~9.2km | 2000-2014 |
| Chlorophyll concentration (minimum) | BO2_chlomin_ss | mg/mü | ~9.2km | 2000-2014 |
| Chlorophyll concentration (range) | BO2_chlorange_ss | mg/mü | ~9.2km | 2000-2014 |
| Current velocity (maximum) | BO2_curvelmax_ss | m/s | ~9.2km | 2000-2014 |
| Current velocity (mean) | BO2_curvelmean_ss | m/s | ~9.2km | 2000-2014 |
| Current velocity (minimum) | BO2_curvelmin_ss | m/s | ~9.2km | 2000-2014 |
| Current velocity (range) | BO2_curvelrange_ss | m/s | ~9.2km | 2000-2014 |
| Primary production (maximum) | BO2_ppmax_ss | g/mü/day | ~9.2km | 2000-2014 |
| Primary production (mean) | BO2_ppmean_ss | g/mü/day | ~9.2km | 2000-2014 |
| Primary production (minimum) | BO2_ppmin_ss | g/mü/day | ~9.2km | 2000-2014 |
| Primary production (range) | BO2_pprange_ss | g/mü/day | ~9.2km | 2000-2014 |
| Sea surface salinity (maximum) | BO2_salinitymax_ss | PSS | ~9.2km | 2000-2014 |
| Sea surface salinity (mean) | BO2_salinitymean_ss | PSS | ~9.2km | 2000-2014 |
| Sea surface salinity (minimum) | BO2_salinitymin_ss | PSS | ~9.2km | 2000-2014 |
| Sea surface salinity (range) | BO2_salinityrange_ss | PSS | ~9.2km | 2000-2014 |
| Bathymetry (minimum) | BO_bathymin | meters | 30 arcsec | 2016 |
| Bathymetry (maximum) | BO_bathymax | meters | 30 arcsec | 2016 |
| Bathymetry (mean) | BO_bathymean | meters | 30 arcsec | 2016 |

* Units: Milligram per area (mg/mü). Meter per second (m/s). Gram per area per time (g/mü/day). Practical Salinity Scale (PSS). Kilometre (km). Second per arc (arcsec)

**Table S3.** (A) Significance of the RDA and the proportion explained by each component of the full model selection. (B) Significance of the environmental variables selected, with an overall significance of the model and variables at p = 0.0001.

(A)

|  | Inertia | Proportion | Rank |
| --- | --- | --- | --- |
| Total | 3.41E+03 | 1.00E+00 |  |
| Conditional | 1.68E+02 | 4.92E-02 | 6 |
| Constrained | 1.20E+02 | 3.53E-02 | 5 |
| Unconstrained | 3.12E+03 | 9.16E-01 | 202 |

(B)

| **Environmental variable** | **p- value** |
| --- | --- |
| Sea surface temperature minimum | 0.001 *** |
| Primary productivity maximum | 0.001 *** |
| Salinity maximum | 0.001 *** |
| Current velocity range | 0.001 *** |
| Current velocity maximum d | 0.001 *** |

**Table S4.** Pairwise F_ST_ values between sites based on putatively adaptive (this study) and neutral (1) datasets for southern Australian common dolphins (*Delphinus delphis*). Upper right, neutral F_ST_ values, and lower left adaptive F_ST_ values, with their significance of the p-values by the B-Y method corrected represented by ***0.0001, **0.001. Acronyms for sites as in Figure 1.

|  | **ALB** | **ESP** | **GAB** | **SG** | **GSV** | **ROB** | **PORT** | **MEL** | **EWP** |
| --- | --- | --- | --- | --- | --- | --- | --- | --- | --- |
| **ALB** | 0 | 0.022*** | 0.026*** | 0.029*** | 0.056*** | 0.025*** | 0.026*** | 0.033*** | 0.047*** |
| **ESP** | 0.084*** | 0 | 0.015*** | 0.017*** | 0.044*** | 0.014*** | 0.015*** | 0.022*** | 0.036*** |
| **GAB** | 0.054*** | 0.023*** | 0 | 0.007*** | 0.034*** | 0.005*** | 0.006*** | 0.012*** | 0.026*** |
| **SG** | 0.057*** | 0.025*** | 0.012*** | 0 | 0.02*** | 0.004*** | 0.003*** | 0.010*** | 0.026*** |
| **GSV** | 0.117*** | 0.084*** | 0.079*** | 0.047*** | 0 | 0.027*** | 0.025*** | 0.029*** | 0.048*** |
| **ROB** | 0.053*** | 0.023*** | 0.006*** | 0.009*** | 0.059*** | 0 | 0.002*** | 0.007*** | 0.023*** |
| **PORT** | 0.054*** | 0.026*** | 0.007*** | 0.009*** | 0.059*** | 0.001 | 0 | 0.005*** | 0.021*** |
| **MEL** | 0.066*** | 0.026*** | 0.011*** | 0.008** | 0.065*** | 0.009*** | 0.008*** | 0 | 0.02*** |
| **EWP** | 0.085*** | 0.060*** | 0.043*** | 0.031*** | 0.080*** | 0.043*** | 0.043*** | 0.029*** | 0 |

**Table S5.** Significance of Gene Ontology (GO) terms for southern Australian common dolphins (*Delphinus delphis*), comparing the full dataset with the putative candidate loci by Fisher’s exact test. Biological Process (BP), Molecular Function (MF), Cellular Component (CC).

|  | **GO.ID** | **Term** | **Annotated** | **Significant** | **Fisher’s p-value** |
| --- | --- | --- | --- | --- | --- |
| BP | GO:0019219 | Regulation of nucleobase-containing compound metabolic process | 86 | 9 | 0.0021 |
|  | GO:0019438 | Aromatic compound biosynthetic process | 111 | 9 | 0.005 |
|  | GO:1901362 | Organic cyclic compound biosynthetic process | 115 | 9 | 0.0052 |
|  | GO:0018130 | Heterocycle biosynthetic process | 111 | 9 | 0.0056 |
|  | GO:0032774 | RNA biosynthetic process | 88 | 9 | 0.0068 |
|  | GO:0009889 | Regulation of biosynthetic process | 84 | 8 | 0.009 |
|  | GO:0071705 | Nitrogen compound transport | 48 | 5 | 0.009 |
|  | GO:0034654 | Nucleobase-containing compound biosynthetic process | 105 | 9 | 0.0105 |
|  | GO:0008284 | Positive regulation of cell proliferation | 5 | 2 | 0.0111 |
|  | GO:0051171 | Regulation of nitrogen compound metabolic process | 100 | 9 | 0.0118 |
|  | GO:0006351 | Transcription, DNA-templated | 88 | 9 | 0.0127 |
|  | GO:0031326 | Regulation of cellular biosynthetic process | 83 | 8 | 0.0142 |
|  | GO:0009059 | Macromolecule biosynthetic process | 138 | 10 | 0.0207 |
|  | GO:0034645 | Cellular macromolecule biosynthetic process | 136 | 10 | 0.0215 |
|  | GO:0044271 | Cellular nitrogen compound biosynthetic process | 133 | 9 | 0.0241 |
|  | GO:0060255 | Regulation of macromolecule metabolic process | 105 | 9 | 0.0255 |
|  | GO:1905114 | Cell surface receptor signaling pathway involved in cell-cell signaling | 6 | 2 | 0.0326 |
|  | GO:0080135 | Regulation of cellular response to stress | 6 | 2 | 0.0406 |
|  | GO:0033036 | Macromolecule localization | 60 | 5 | 0.0536 |
|  | GO:0070647 | Protein modification by small protein co. | 16 | 2 | 0.0543 |
| MF | GO:0030246 | Carbohydrate binding | 25 | 5 | 0.02 |
|  | GO:0001067 | Regulatory region nucleic acid binding | 12 | 3 | 0.025 |
|  | GO:0008234 | Cysteine-type peptidase activity | 22 | 3 | 0.027 |
|  | GO:0044212 | Transcription regulatory region DNA bind. | 12 | 3 | 0.05 |
|  | GO:0005506 | Iron ion binding | 17 | 3 | 0.079 |
|  | GO:1990837 | Sequence-specific double-stranded DNA bi. | 9 | 2 | 0.083 |
|  | GO:0048037 | Cofactor binding | 47 | 6 | 0.083 |
|  | GO:0016491 | Oxidoreductase activity | 67 | 7 | 0.102 |
|  | GO:0043169 | Cation binding | 297 | 25 | 0.109 |
|  | GO:0008092 | Cytoskeletal protein binding | 72 | 6 | 0.112 |
|  | GO:0060089 | Molecular transducer activity | 68 | 7 | 0.115 |
|  | GO:0004497 | Monooxygenase activity | 6 | 2 | 0.115 |
|  | GO:0016705 | Oxidoreductase activity, acting on paire. | 13 | 3 | 0.127 |
|  | GO:0016758 | Transferase activity, transferring hexos. | 13 | 2 | 0.148 |
|  | GO:0031406 | Carboxylic acid binding | 10 | 2 | 0.153 |
|  | GO:0043177 | Organic acid binding | 10 | 2 | 0.157 |
|  | GO:0005319 | Lipid transporter activity | 5 | 1 | 0.171 |
|  | GO:0005488 | Binding | 845 | 56 | 0.171 |
|  | GO:0008135 | Translation factor activity, RNA binding | 12 | 1 | 0.176 |
|  | GO:0008081 | Phosphoric diester hydrolase activity | 15 | 2 | 0.176 |
| CC | GO:0030054 | Cell junction | 14 | 3 | 0.045 |
|  | GO:0042995 | Cell projection | 17 | 3 | 0.057 |
|  | GO:0030117 | Membrane coat | 9 | 2 | 0.074 |
|  | GO:0031410 | Cytoplasmic vesicle | 13 | 2 | 0.1 |
|  | GO:0048475 | Coated membrane | 9 | 2 | 0.108 |
|  | GO:0031982 | Vesicle | 13 | 2 | 0.121 |
|  | GO:0097708 | Intracellular vesicle | 13 | 2 | 0.127 |
|  | GO:0005929 | Cilium | 12 | 2 | 0.131 |
|  | GO:0031514 | Motile cilium | 5 | 2 | 0.152 |
|  | GO:0044433 | Cytoplasmic vesicle part | 6 | 1 | 0.194 |
|  | GO:0098796 | Membrane protein complex | 57 | 5 | 0.216 |
|  | GO:0044429 | Mitochondrial part | 7 | 1 | 0.223 |
|  | GO:0044456 | Synapse part | 16 | 2 | 0.243 |
|  | GO:0016020 | Membrane | 577 | 37 | 0.243 |
|  | GO:0005730 | Nucleolus | 8 | 1 | 0.27 |
|  | GO:1904949 | ATPase complex | 6 | 1 | 0.274 |
|  | GO:0044425 | Membrane part | 535 | 34 | 0.296 |
|  | GO:0044451 | Nucleoplasm part | 16 | 1 | 0.308 |
|  | GO:0012505 | Endomembrane system | 56 | 4 | 0.357 |
|  | GO:0097458 | Neuron part | 8 | 1 | 0.361 |

**Table S6.** Function of the candidate genes found in exonic regions, which were over enriched by the Gene Ontology analyses, for the 747 putatively adaptive SNPs discovered by the RDA of southern Australian common dolphins (*Delphinus delphis*).

| **Genes in exons** | **Environmental variable associated** | **Pathway (reactome)** | **Function**  **(Uniprot)** | **Family** | **General function described** | **References** |
| --- | --- | --- | --- | --- | --- | --- |
| **MAN2B1**  Missense exon | RDA primary productivity max | Lysosome lumen: Lysosomal oligosaccharide catabolism mainly. | *Lipotes vexillifer* (Yangtze river dolphin) Protein Alpha-mannosidae.  Human: Necessary for the catabolism of N-linked carbohydrates released during glycoprotein turnover. | MAN | Breaks complex sugar molecules “more energy” in the carbohydrate metabolism | (5-8) |
| **NR2F2**  Missense  exon | RDA salinity max | Nucleoplasm- NR2F6:Gene expression in transcription pathway. Adipogenesis is the process of cell differentiation by which preadipocytes become adipocytes. | *Lipotes vexillifer* (Yangtze river dolphin) NR2F2-gene and COUP transcription factor 2-like isoform X3.  Human: NR2F2 Ligand-activated transcription factor. | NR2 | Regulation of adipogenesis, glucose, homeostasis and metabolism energy | (9-11) |
| **ZPF57**  Synonymous exon | RDA primary productivity max | ZPF: Nucleoplasm- Gene expression in transcription: generic transcriptions pathway, genes and factors involve in megakaryocyte differentiation, metabolism mRNA stability by proteins. | *Physeter macrocephalus* (Sperm whale). ZPF57-gene, zinc finger protein 57 homolog. Molecular function: metal ion binding and nucleic acid binding. Biological process: regulation of transcription DNA-templated.  Human: Transcription regulator required to maintain maternal and paternal gene imprinting. | Part of the large group of ZPF | Acts by controlling DNA methylation during earliest multicellular stages of development and may be altered by nutrients in the diet. | (12-16) |

**Figure S1.** Multicollinearity between the five environmental variables used for the RDA. Salinity maximum (BO2_salinitymax_ss), primary productivity maximum (BO2_ppmax_ss), sea surface temperature minimum (BO_sstmin), current velocity maximum (BO2_curvelmax_ss) and current velocity range (BO2_curvelrange_ss). Numbers in the upper right matrix are the correlation values of each comparison; the smaller the number gets, the closest to zero the correlation between the variables is. Variables were standardised from 0-1.

**Figure S2.** Akaike Information Criterion (AIC) used to determine the best-supported number of clusters in the PCA, based on the putative adaptive dataset of southern Australian common dolphins (*Delphinus delphis*).

(A) K2


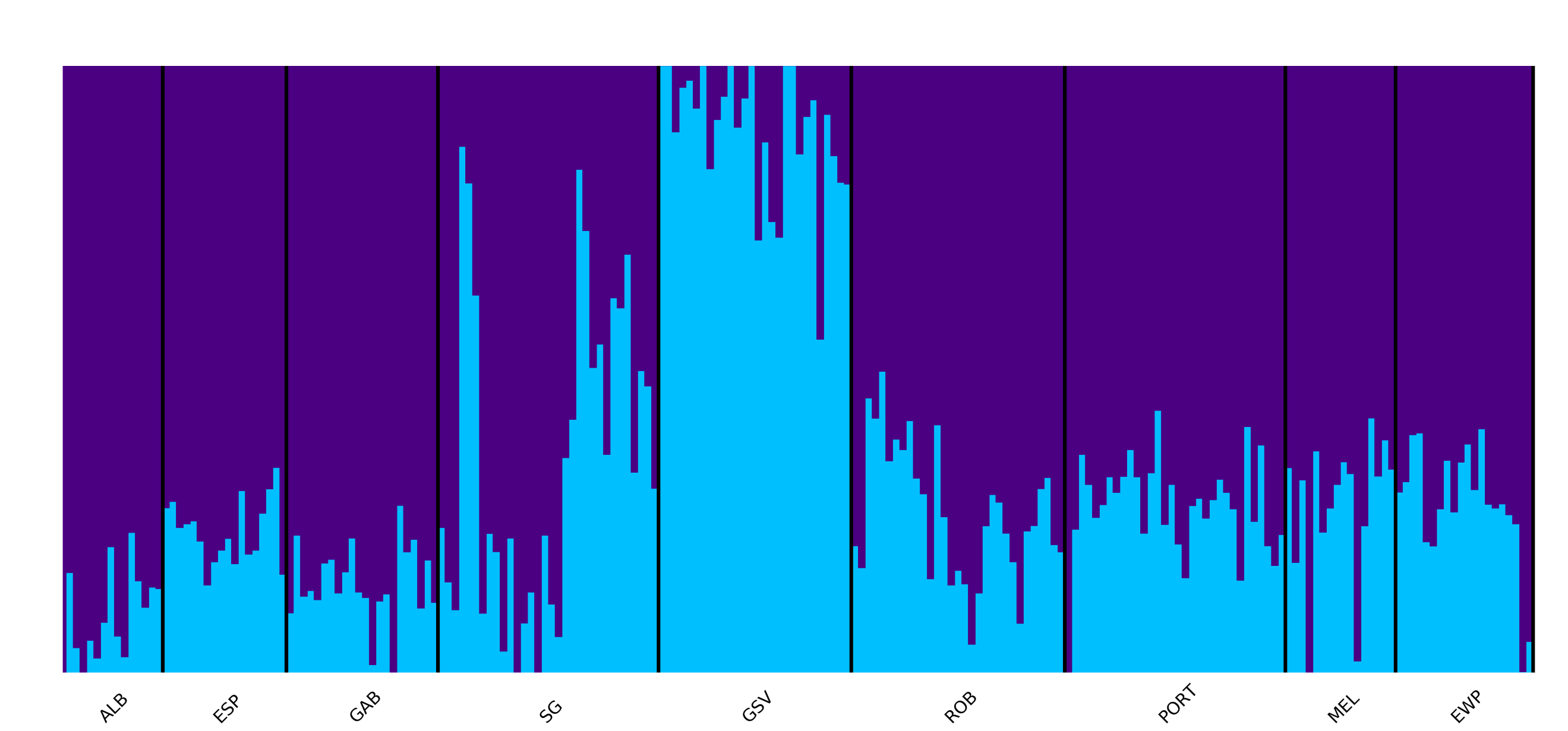


(B) K3


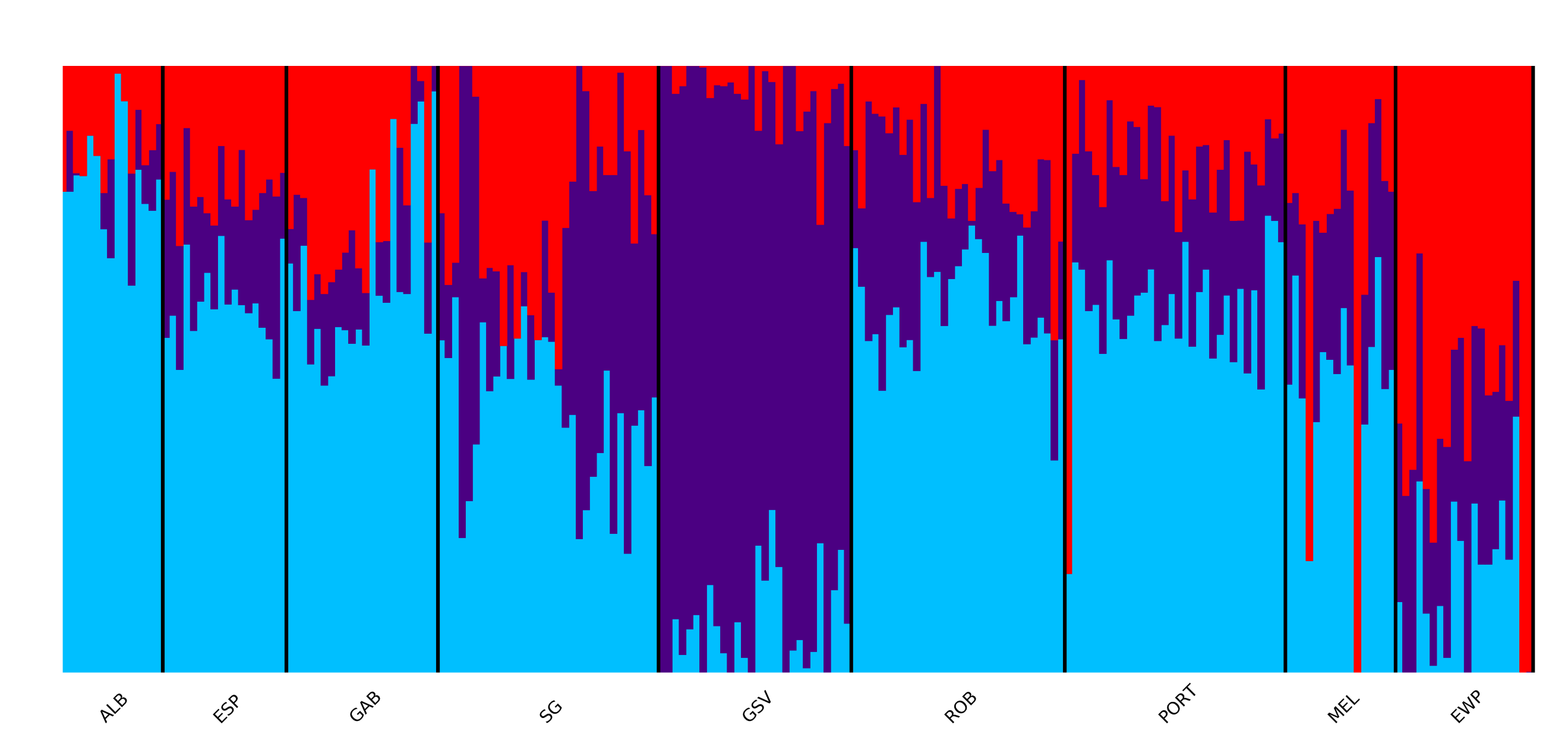


(C) K4*


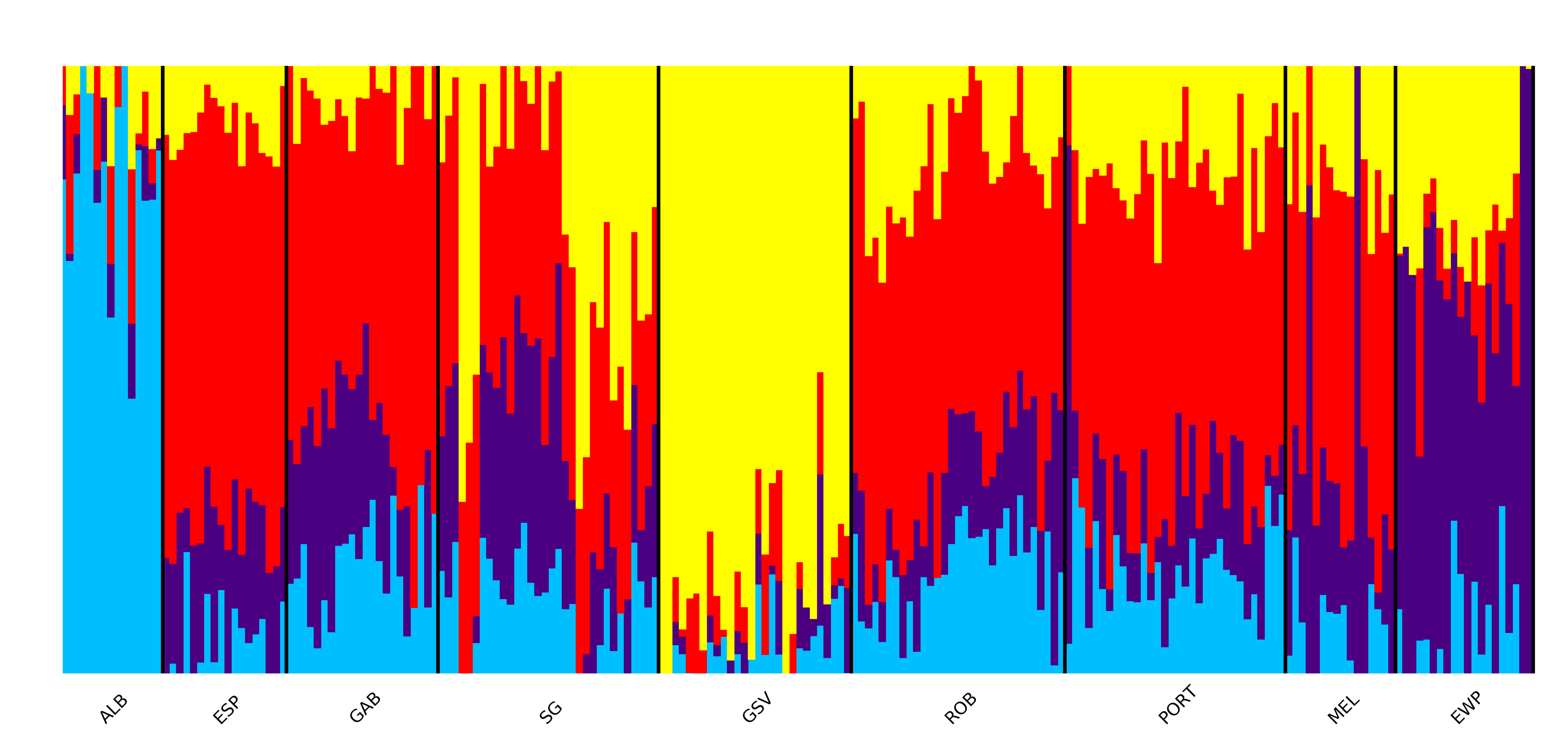


(D) K5*


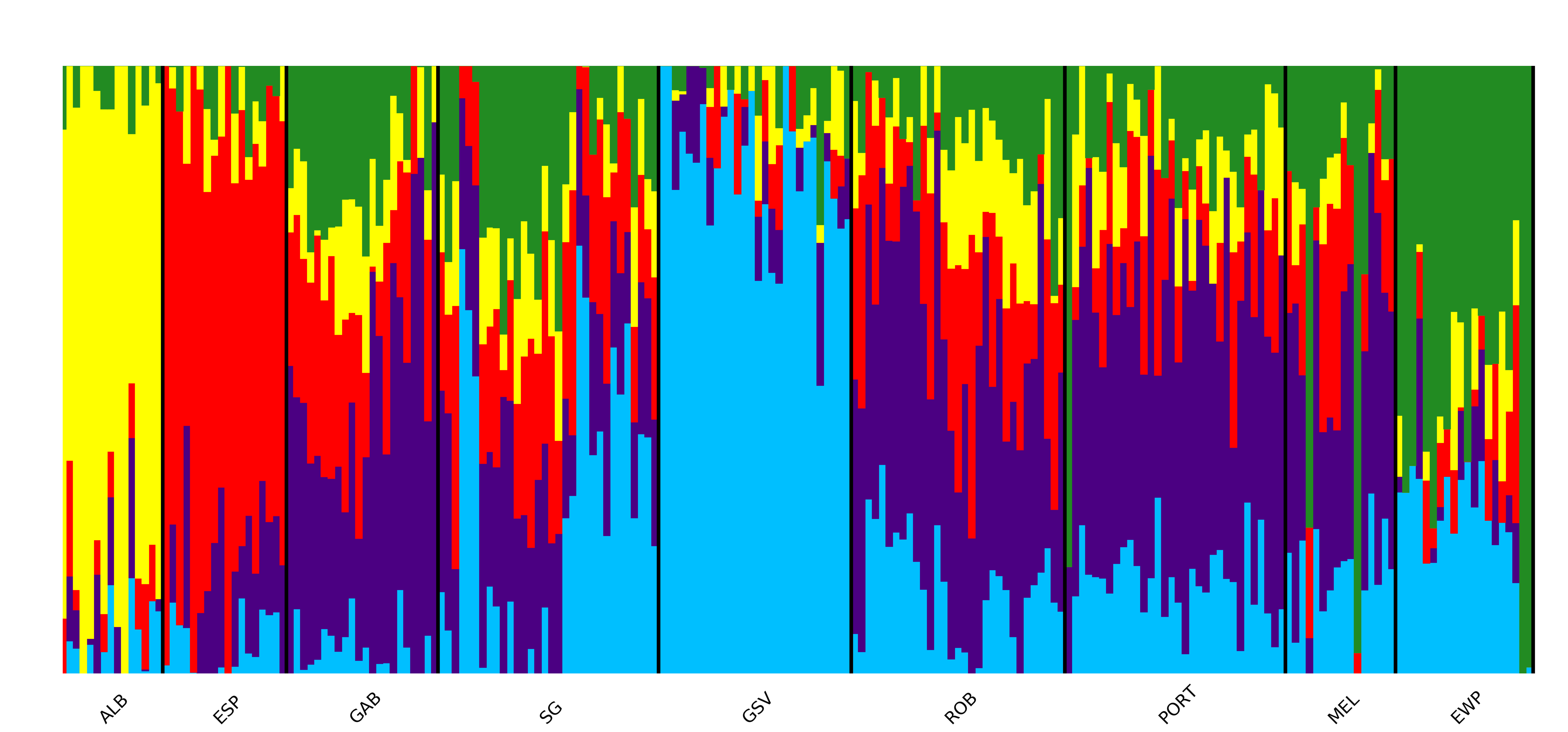


(E) K6
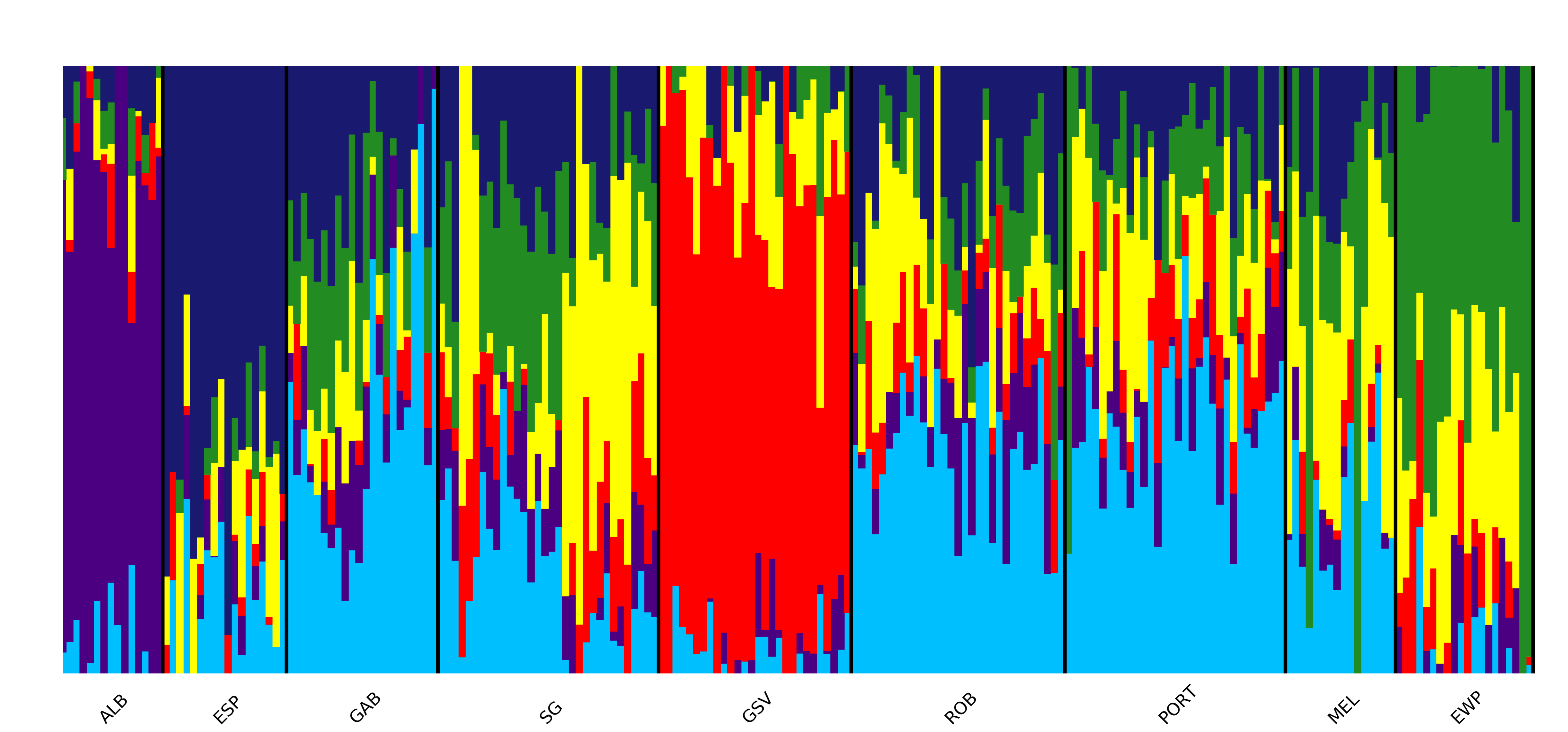
(F) K7


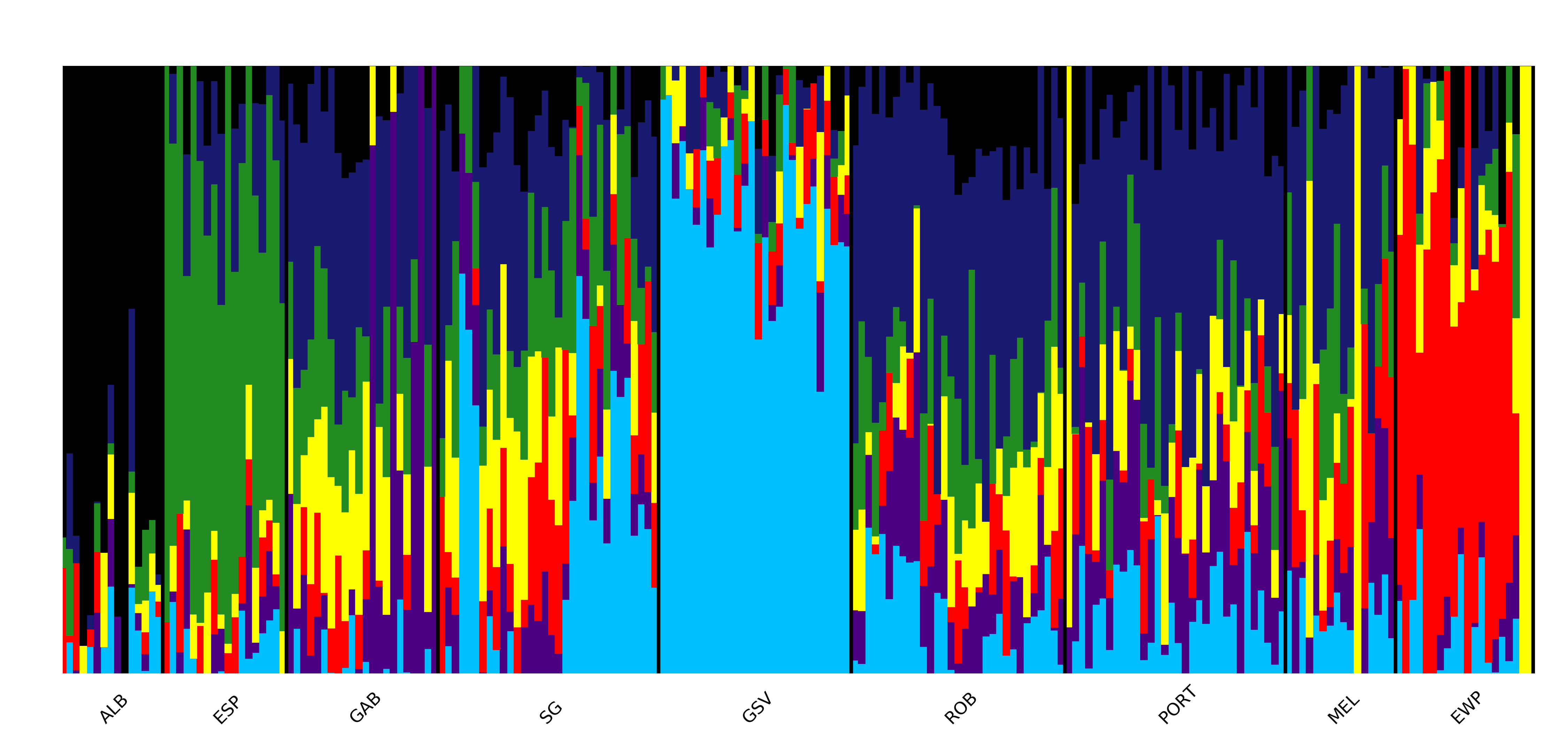


**Figure S3.** Population genomic structure analysis using Admixture based on the putatively adaptive SNPs for southern Australian common dolphins (*Delphinus delphis*) (labelled by sampling site and individual). K represents the number of populations tested (A to F), in which K4* and K5* are both correctly assigned as they are the most supported and highly likely number of local populations suggested by the analyses. Acronyms for sites as in Figure 1.

**(A)**

**(B)**

**(C)**

**Figure S4.** Principal Component Analysis (PCA) based on 747 candidate adaptive loci for southern Australian common dolphins (*Delphinus delphis*). **(A)** Explanatory axes PC1 vs PC2. **(B)** Explanatory axes PC1 vs PC3. **(C)** Explanatory axes PC2 vs PC3. Acronyms for sites as in Figure 1.

**Figure S5.** Heatmap of pairwise F_ST_ values between sites based on the adaptive (this study) and neutral (1) SNP datasets for southern Australian common dolphins (*Delphinus delphis*). Upper right, neutral dataset, and lower left, adaptive dataset. Acronyms for sites as in Figure 1.


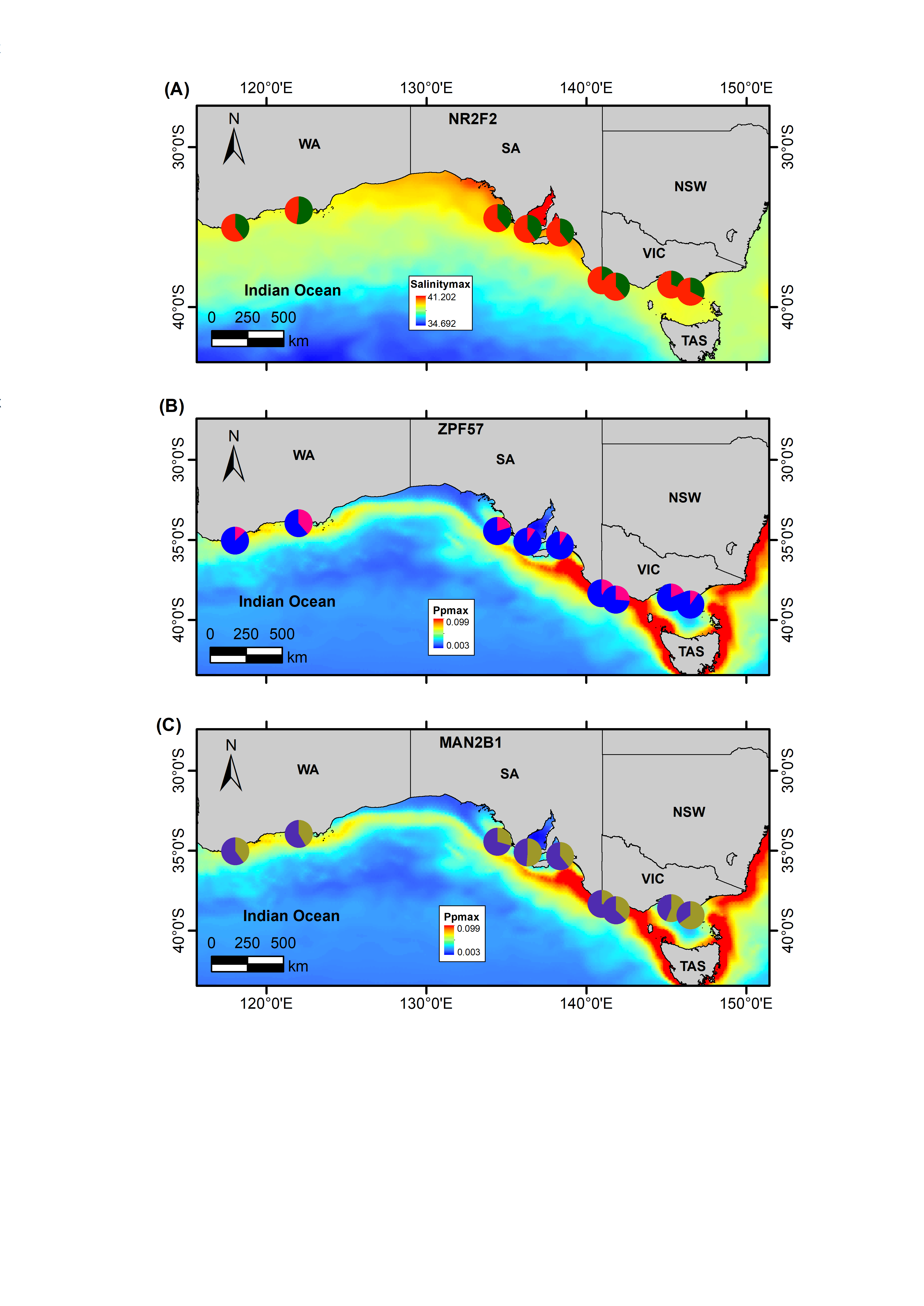


**Figure S6.** Allele frequency changes for the candidate gene variants found in exonic regions of common dolphins (*Delphinus delphis*) across sampling sites in southern Australia. **(A)** NR2F6/NR2F2 was associated with maximum salinity, **(B)** ZFP57, with primary productivity maximum, and **(C)** MAN2B1, with primary productivity maximum.

**Figure S7.** Minor allele frequencies of 26 SNPs selected by a gene enrichment test, with variants in coding or non-coding regions.

**References**

1. Barceló A, Sandoval-Castillo J, Stockin KA, Bilgmann K, Attard CRM, Zanardo N, et al. A Matter of Scale: Population Genomic Structure and Connectivity of Fisheries At-Risk Common Dolphins (*Delphinus delphis*) From Australasia. Frontiers in Marine Science. 2021;8.

2. Linck E, Battey CJ. Minor allele frequency thresholds strongly affect population structure inference with genomic data sets. Mol Ecol Resour. 2019;19(3):639-47.

3. Tyberghein L, Verbruggen H, Pauly K, Troupin C, Mineur F, De Clerck O. Bio-ORACLE: a global environmental dataset for marine species distribution modelling. Global Ecology and Biogeography. 2012;21(2):272-81.

4. Assis J, Tyberghein L, Bosch S, Verbruggen H, Serrão EA, De Clerck O. Bio-ORACLE v2.0: Extending marine data layers for bioclimatic modelling. Global Ecology and Biogeography. 2018;27(3):277-84.

5. Dineshram R, Q Q, Sharma R, Chandramouli K, Yalamanchili HK, Chu I, et al. Comparative and quantitative proteomics reveal the adaptive strategies of oyster larvae to ocean acidification. Proteomics. 2015;15(23-24):4120-34.

6. Lazzarotto V. Consequences of long-term feeding trout with plant-based diets on the regulation of energy and lipid metabolism: special focus on trans-generational effects and early stages.: L’ Universite de Pau et des Pay Del'Adour; 2016.

7. Sproles AE, Oakley CA, Matthews JL, Peng L, Owen JG, Grossman AR, et al. Proteomics quantifies protein expression changes in a model cnidarian colonised by a thermally tolerant but suboptimal symbiont. The ISME Journal. 2019;13(9):2334-45.

8. Tivey TR, Parkinson JE, Mandelare PE, Adpressa DA, Peng W, Dong X, et al. N-Linked Surface Glycan Biosynthesis, Composition, Inhibition, and Function in Cnidarian-Dinoflagellate Symbiosis. Microb Ecol. 2020;80(1):223-36.

9. Baldwin WS, Boswell WT, Ginjupalli G, Litoff EJ. Annotation of the Nuclear Receptors in an Estuarine Fish species, *Fundulus heteroclitus*. Nucl Receptor Res. 2017;4.

10. Jeong BC, Kang IH, Koh JT. MicroRNA-302a inhibits adipogenesis by suppressing peroxisome proliferator-activated receptor gamma expression. FEBS Lett. 2014;588(18):3427-34.

11. Li L, Xie X, Qin J, Jeha GS, Saha PK, Yan J, et al. The nuclear orphan receptor COUP-TFII plays an essential role in adipogenesis, glucose homeostasis, and energy metabolism. Cell Metab. 2009;9(1):77-87.

12. Amarasekera M, Martino D, Ashley S, Harb H, Kesper D, Strickland D, et al. Genome-wide DNA methylation profiling identifies a folate-sensitive region of differential methylation upstream of ZFP57-imprinting regulator in humans. FASEB J. 2014;28(9):4068-76.

13. Irwin RE, Thursby SJ, Ondicova M, Pentieva K, McNulty H, Richmond RC, et al. A randomized controlled trial of folic acid intervention in pregnancy highlights a putative methylation-regulated control element at ZFP57. Clin Epigenetics. 2019;11(1):31.

14. Zglejc K, Franczak A. Peri-conceptional under-nutrition alters the expression of TRIM28 and ZFP57 in the endometrium and embryos during peri-implantation period in domestic pigs. Reprod Domest Anim. 2017;52(4):542-50.

15. Foote AD, Vijay N, Avila-Arcos MC, Baird RW, Durban JW, Fumagalli M, et al. Genome-culture coevolution promotes rapid divergence of killer whale ecotypes. Nat Commun. 2016;7:11693.

16. Yim HS, Cho YS, Guang X, Kang SG, Jeong JY, Cha SS, et al. Minke whale genome and aquatic adaptation in cetaceans. Nat Genet. 2014;46(1):88-92.
